# Patterns of cell cycle checkpoint deregulation associated with intrinsic molecular subtypes of human breast cancer cells

**DOI:** 10.1038/s41523-017-0009-7

**Published:** 2017-03-31

**Authors:** Jacquelyn J. Bower, Leah D. Vance, Matthew Psioda, Stephanie L. Smith-Roe, Dennis A. Simpson, Joseph G. Ibrahim, Katherine A. Hoadley, Charles M. Perou, William K. Kaufmann

**Affiliations:** 1 Department of Pathology and Laboratory Medicine, University of North Carolina at Chapel Hill, Chapel Hill, NC 27599 USA; 2 Lineberger Comprehensive Cancer Center, University of North Carolina at Chapel Hill, Chapel Hill, NC 27599 USA; 3 Department of Biostatistics, University of North Carolina at Chapel Hill, Chapel Hill, NC 27599 USA; 4 Center for Environmental Health and Susceptibility, University of North Carolina at Chapel Hill, Chapel Hill, NC 27599 USA; 5 Department of Genetics, University of North Carolina at Chapel Hill, Chapel Hill, NC 27599 USA; 6 Division of the National Toxicology Program, National Institute of Environmental Health Sciences, 111 T.W. Alexander Drive, National Institutes of Health, Research Triangle Park, NC 27709 USA

## Abstract

Genomic instability is a hallmark of breast cancer, contributes to tumor heterogeneity, and influences chemotherapy resistance. Although Gap 2 and mitotic checkpoints are thought to prevent genomic instability, the role of these checkpoints in breast cancer is poorly understood. Here, we assess the Gap 2 and mitotic checkpoint functions of 24 breast cancer and immortalized mammary epithelial cell lines representing four of the six intrinsic molecular subtypes of breast cancer. We found that patterns of cell cycle checkpoint deregulation were associated with the intrinsic molecular subtype of breast cancer cell lines. Specifically, the luminal B and basal-like cell lines harbored two molecularly distinct Gap 2/mitosis checkpoint defects (impairment of the decatenation Gap 2 checkpoint and the spindle assembly checkpoint, respectively). All subtypes of breast cancer cell lines examined displayed aberrant DNA synthesis/Gap 2/mitosis progression and the basal-like and claudin-low cell lines exhibited increased percentages of chromatid cohesion defects. Furthermore, a decatenation Gap 2 checkpoint gene expression signature identified in the cell line panel correlated with clinical outcomes in breast cancer patients, suggesting that breast tumors may also harbor defects in decatenation Gap 2 checkpoint function. Taken together, these data imply that pharmacological targeting of signaling pathways driving these phenotypes may lead to the development of novel personalized treatment strategies for the latter two subtypes which currently lack targeted therapeutic options because of their triple negative breast cancer status.

## Cell cycle: Breast cancer subtypes exhibit distinct checkpoint dysfunctions

Unique subtypes of breast cancer display distinctive patterns of genomic instability that affect cell division. Jacquelyn J. Bower and colleagues at the University of North Carolina at Chapel Hill, USA, assessed the molecular function of several G2-M cell cycle checkpoints—which ensure that cells don’t begin mitotic cell division until DNA replication is complete and they have had a chance to repair any damaged DNA—in 24 breast cancer and mammary epithelial cell lines that collectively represent four of the six intrinsic molecular subtypes of breast cancer. The distinct patterns of cell cycle checkpoint deregulation that the researchers found suggest that pharmacological targeting of the dysfunction in each type of breast cancer could lead to personalized treatment strategies. The findings also point to diagnostic biomarkers that could help predict patient responses to novel drugs or existing chemotherapies.

## Introduction

**Fig. 1:**
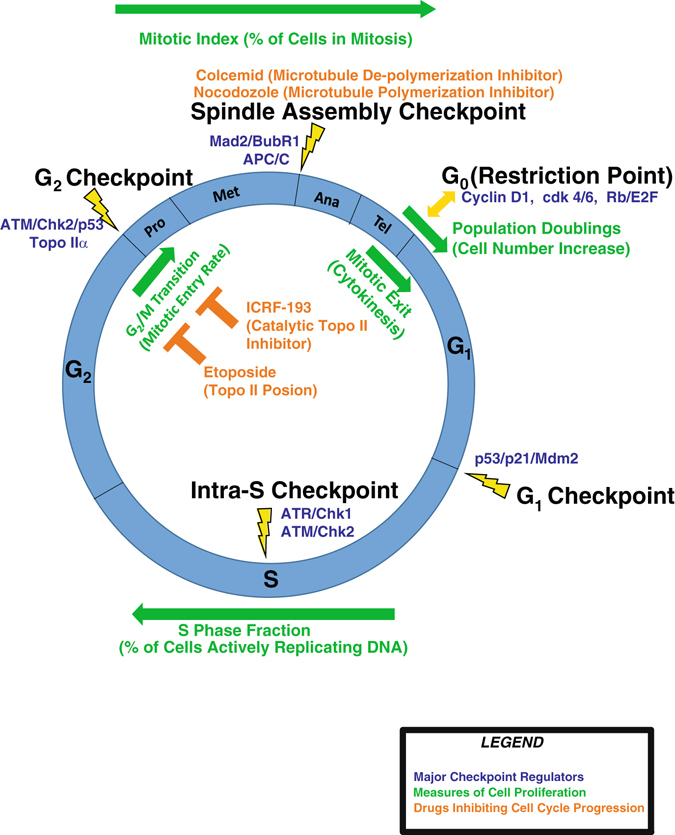
Diagram of cell cycle regulation. Phases of the cell cycle are shown inside the blue circle in the center of the figure (G_0_, G_1_, S, G_2_, and mitosis which consists of several sub-phases: prophase (Pro), metaphase (Met), anaphase (Ana), and telophase (Tel)). The G_0_ Restriction Point is designated with a *yellow dual headed arrow* to illustrate the reversible nature of cell cycle entry and quiescence. As cells progress through the cycle, exogenous perturbations can activate checkpoints that arrest cells during phase transitions (checkpoints are designated by *yellow lightning bolts*). Several measures of cellular proliferation are shown in green and span the cell cycle phases in which these markers are present. Drugs that inhibit cell cycle progression are shown in orange with their targets and mechanisms of action designated in subsequent parentheses. Components of major regulatory pathways triggering each checkpoint are listed in *dark blue font* near the checkpoint in which they play a role. Precise control over the regulation of the cell cycle is a requirement for ensuring accurate DNA replication and cell division

Cellular division is controlled by a tightly regulated process that requires accurate separation of sister chromatids upon the completion of DNA replication in order to produce two genetically identical daughter cells. The regulatory signals that control cell division are collectively referred to as the cell cycle, which is comprised of five distinct phases: quiescence (G_0_), Gap 1 (G_1_), DNA replication/synthesis (S), Gap 2 (G_2_), and mitosis (M) (Fig. 1). Transitions between different phases of the cell cycle are induced via oscillating levels of cyclins and cyclin-dependent kinases (cdks); each phase of the cell cycle is characterized by the formation of specific complexes of cyclin/cdk heterodimers.

Intracellular and/or external stimuli can halt progression of the cell cycle through a complex network of signaling events that interfere with cyclin/cdk activities controlling cell cycle progression. This pause in cell cycle progression is often referred to as a “checkpoint” and allows the cell time to repair damaged DNA or acquire sufficient levels of growth factors before transitioning to the next phase; if the DNA damage is too severe to repair, the cell may activate apoptotic signaling cascades to prevent the transmission of damaged DNA to its daughter cells. Thus, cell cycle checkpoints ensure ordered progression of the cell cycle, are critical for maintaining genomic stability, act as barriers to carcinogenesis, and are often deregulated in tumors.^1–3^

At least four cell cycle checkpoints may be deregulated in cancer cells: the restriction point (G_0_/G_1_), the G_1_ checkpoint, the G_2_ checkpoint, and the mitosis-associated spindle assembly checkpoint (SAC). The G_0_/G_1_ restriction point is the point in G_1_ at which the withdrawal of growth factors no longer induces reversion to a quiescent state; thus, it controls the cell’s commitment to division.^4^ The restriction checkpoint is largely controlled by the Rb/E2F signaling pathway: release of E2F transcription factors from Rb allows E2F to transcriptionally activate genes that promote the initiation of DNA replication and S phase entry. Both overexpression of upstream regulators of Rb (such as Cyclin D1/cdk4/6) or inactivating mutations in the *RB1* gene can degrade this checkpoint and lead to early activation of DNA replication.^5, 6^ The G_1_ DNA damage checkpoint delays the initiation of DNA replication in the presence of DNA damage and is largely controlled by the p53/p21/Mdm2 pathway.^7, 8^ Loss of this checkpoint can often occur via *TP53* mutations or inactivation of wild-type p53 by viral proteins and lead to error-prone DNA synthesis due to the diminished amount of time allotted for DNA repair.^9, 10^

Two types of G_2_ checkpoint responses have been previously identified: the DNA damage G_2_ checkpoint and the decatenation G_2_ checkpoint.^11–13^ The DNA damage G_2_ checkpoint delays the initiation of mitosis upon DNA damage by sequestering inactive cyclin B1/cdk1 in the cytoplasm, thus preventing entry into mitosis.^14, 15^ The decatenation G_2_ checkpoint is molecularly distinct from the DNA damage G_2_ checkpoint in that it is activated in response to catalytic inhibition of topoisomerase IIα (topo IIα) without overt DNA damage.^12, 16, 17^ Major players in both the DNA damage and decatenation G_2_ checkpoint include the ATM/Chk2/p53 pathway and attenuation of either G_2_ checkpoint leads to chromosomal instability.^18^ Finally, the SAC acts in mitosis to delay the onset of anaphase until all chromosomes exhibit bipolar attachment to the mitotic spindle.^2, 19, 20^ Major effectors of SAC function include APC/C, BubR1, and Mad2, and impaired SAC signaling often leads to the formation of multipolar spindles and/or the unequal partitioning of sister chromatids into daughter cells.^21^ Defects in all of these cell cycle checkpoints have been shown to play an integral role in tumor initiation and/or progression and affect the sensitivity of tumors to both cytotoxic and endocrine drugs.^22–24^

The role of the restriction and G_1_ checkpoints in breast cancer signaling, genome maintenance, and chemosensitivity has been previously characterized;^24, 25^ however, the relationship between G_2_ or SAC checkpoint function, genomic instability, and chemoresistance is poorly understood. Six intrinsic molecular subtypes of breast tumors have been proposed,^26, 27^ and several studies imply that these subtypes contain similar molecular defects that contribute to disease progression and variability in chemotherapeutic response,^28-30^ which may confer synthetic lethality to subsets of cancer cells.^31, 32^ Although these studies imply that genomic instability plays a role in these processes, the nature of the specific molecular defects contributing to breast cancer outcomes remains elusive. To explore the underlying mechanisms of genomic instability in breast cancer cells, G_2_ and M checkpoint functions were assessed in a panel of 24 cell lines using a flow cytometry-based mitotic entry rate (MER) assay that allows for discrimination among three molecularly distinct G_2_/M checkpoints: the DNA damage G_2_ checkpoint, the decatenation G_2_ checkpoint, and the SAC. The panel is comprised of cell lines representing four of the six intrinsic molecular subtypes of breast tumors: six luminal B (LumB), four basal-like (BL), six claudin-low (CL), and four Her2-enriched (Her2E) cell lines. Luminal A and normal-like breast cancer cell lines are unavailable, and four immortalized non-tumorigenic human mammary epithelial cell lines (HMECs) were employed as positive controls. These cell lines recapitulate the gene expression profiles, abnormal genomic features, and phenotypes of in vivo breast tumors,^33-35^ and exhibit subtype-specific responses to several chemotherapeutic agents.^32^

Because no comprehensive functional analysis of G_2_/M checkpoints has been described for breast cancer cell lines to date and existing reports almost exclusively utilize three breast cancer cell lines (MCF7, HCC1937, and MDA-MB-231) to investigate the accumulation of cells in G_2_/M (but not checkpoint function), the data generated from this study provides a valuable resource for delineating the role of G_2_/M checkpoints in breast cancer cells. Furthermore, the findings of this study indicate that deregulation of G_2_ and M checkpoint functions, aberrant cell cycle regulation patterns, and chromatid cohesion defects coincide with breast cancer intrinsic molecular subtypes and that a gene signature for decatenation G_2_ checkpoint function identified in the cell line panel correlates with clinical outcomes in breast cancer patients. These data collectively suggest that pharmacological targeting of pathways driving these genomic instability phenotypes may lead to the development of new personalized treatment strategies for breast cancer subtypes that currently lack targeted therapies.

## Results

### The decatenation G_2_ checkpoint is impaired in LumB breast cancer cell lines

To assess G_2_/M checkpoint function, a MER assay utilizing two topo II inhibitors exhibiting distinct mechanisms of action was employed to monitor the G_2_-M transition rate. Cells were incubated with colcemid (to prevent mitotic exit) for 2–6 h in the presence or absence of the topo II catalytic inhibitor ICRF-193 (which does not overtly damage DNA) to measure decatenation G_2_ checkpoint function or the topo II poison etoposide (which induces DNA damage) to measure DNA damage G_2_ checkpoint function at concentrations that arrest 98–100% of normal human diploid fibroblasts (NHF1-hTERTs) and immortalized lymphoblasts in G_2_.^36^ The use of colcemid in the MER assay blocks cells from exiting mitosis, allowing for a strict examination of the G_2_ to M transition, thus minimizing any confounding effects related to the rate of mitotic exit. The MER was calculated from the linear portion of the resulting line (2–6 h time points) and is expressed as the percentage of cells entering mitosis per hour.^36^

**Fig. 2:**
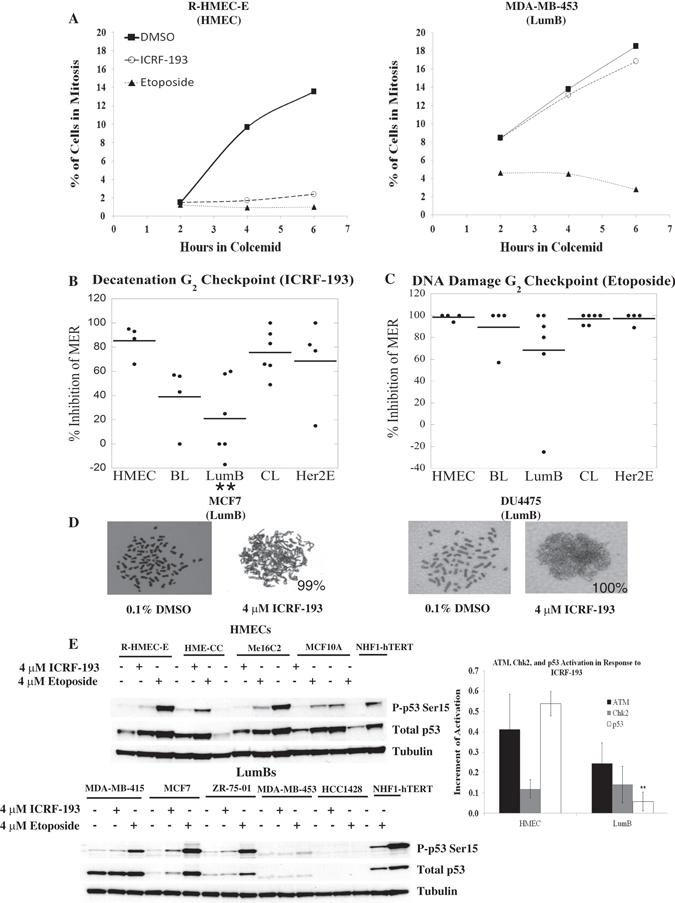
The decatenation G_2_ checkpoint response is impaired in luminal B (LumB) breast cancer cell lines. A panel of non-tumorigenic immortalized mammary epithelial cell lines (HMEC) and breast cancer cell lines were assessed for G_2_ and M checkpoint functions using a mitotic entry rate (MER) assay to monitor the rate of the G_2_/M transition in the presence of the topo II catalytic inhibitor ICRF-193 (decatenation G_2_ checkpoint) or the DNA-damaging topo II poison etoposide (DNA damage G_2_ checkpoint). **a** Example MERs of an HMEC (R-HMEC-E) cell line with effective decatenation and DNA damage G_2_ checkpoints and a LumB (MDA-MB-453) cell line with a defective decatenation G_2_ checkpoint. **b** and **c** The average percent inhibition of the MER of each cell line grouped according to intrinsic molecular subtype. Each point on the graph represents the average of 3 independent experiments for an individual cell line, and the bold lines represent the class average. **d** Example metaphases of LumB cell lines in the presence of DMSO exhibiting individualized chromosomes. Severely under-condensed and/or entangled chromosomes were observed in >88% of LumB cells upon treatment with 4 µM ICRF-193, suggesting that ICRF-193 is capable of inhibiting topoisomerase II in LumB cell lines. Percentages of entangled/under-condensed chromosomes are shown in the lower right hand corner of each ICRF-193 treated example. **e** HMECs activate p-Ser15 p53 after ICRF-193 or etoposide treatment (top panel). The HME-CC etoposide sample displayed aberrant mobility of ATM and reduced expression of p53 which could not be reproduced; therefore, this sample was omitted from the analysis. LumB cell lines exhibit reduced levels of p-Ser15 p53 in response to ICRF-193 (lower panel). Quantification of p53 activation is shown in the right panel. Data are representative of two independent experiments. *p-value < 0.05, **p-value that remains significant when controlling for FDR (5%), BL: basal-like, CL: claudin-low, Her2E: Her2-enriched

MER examples are shown for the HMEC cell line R-HMEC-E in the left panel of Fig 2a. These cells accumulated in mitosis when incubated in colcemid (and the vehicle control dimethyl sulfoxide (DMSO), closed squares) reflecting their transition from G_2_ to M; however, in the presence of the catalytic inhibitor ICRF-193 (open circles), mitotic accumulation of R-HMEC-E cells was severely inhibited suggesting that the majority of these cells activate a decatenation G_2_ checkpoint response. In the presence of the topo II poison etoposide, the mitotic accumulation of R-HMEC-E cells was also severely inhibited (closed triangles), indicating that this cell line exhibits an effective DNA damage G_2_ checkpoint. Conversely, the LumB breast cancer cell line MDA-MB-453 continued to accumulate in mitosis in the presence of ICRF-193, but not etoposide (Fig. 2a, *right panel*), suggesting that a majority of these cells evaded the decatenation G_2_ checkpoint but arrested in G_2_ upon DNA damage.

All 24 cell lines were subjected to the MER-based G_2_ checkpoint assay and the averages of three independent experiments for each cell line (grouped according to molecular subtype) are shown in Fig. 2b (individual cell line averages ± SEM are provided in Table S1). As expected, all four positive control HMEC cell lines displayed severe MER inhibition in response to ICRF-193 suggesting that this class exhibits an effective decatenation G_2_ checkpoint. In contrast, checkpoint functions of the breast cancer cell lines were highly variable (Fig. 2b). Upon grouping the cell lines by intrinsic molecular subtype, the LumB and BL cell lines appeared to exhibit attenuated decatenation G_2_ checkpoint function, whereas the HER2E and CL subtypes displayed checkpoint function similar to the HMECs. A set of statistical linear mixed models (LMMs) was employed to compare G_2_ checkpoint functions among the different subtypes (see Supplementary Information, (SI)). These analyses established that the LumB class (but not the BL class) of breast cancer cell lines exhibited defects in decatenation G_2_ checkpoint function (**p < 0.05, FDR < 0.05). Although the distribution of % inhibition of MER in the BL cell lines initially appeared similar to that of the LumB group, closer inspection of the MER data revealed that the BL group exhibited very low MERs in the absence of a topo II catalytic inhibitor, indicating that this group was not responding to colcemid treatment; thus, the percent inhibition of MER appears artificially low in the BL group.

Complementary experiments were performed to assess whether breast cancer cell lines were capable of activating the DNA damage G_2_ checkpoint using a concentration of the topo II poison etoposide equivalent to that of the catalytic inhibitor ICRF-193 (4 µM). All four HMEC cell lines displayed an effective DNA damage G_2_ checkpoint in response to etoposide (Fig. 2c). The majority of the breast cancer cell lines also exhibited large MER inhibition in the presence of etoposide and no significant differences were observed for any of the breast cancer cell line classes; thus, these breast cancer cell lines were capable of activating the DNA damage G_2_ checkpoint at levels comparable to those of HMECs, with the exception of the DU4475 LumB cell line (Table S1).

To confirm that ICRF-193 was able to inhibit topo II activity in the LumB cell lines, cytogenetic preparations were examined in the presence or absence of ICRF-193 and representative metaphases of two LumB cell lines are shown in Fig. 2d. In the presence of DMSO, the majority of the LumB metaphases exhibited individualized condensed chromosomes. However, upon inhibition of topo II with ICRF-193, >88% of all LumB mitotic cells displayed chromosomes that were under-condensed and/or entangled (data not shown). Thus, the observed defect in decatenation G_2_ checkpoint function was unlikely to be the result of a failure of ICRF-193 to inhibit topo II catalytic activity in the LumB class, supporting the conclusion that the defect is due to dysfunctional checkpoint activation and not an inability of the LumB class to respond to ICRF-193 treatment.

Previous studies demonstrate that p53 is activated in response to ICRF-193, and cell lines with defective decatenation G_2_ checkpoint function exhibit attenuated activation of p53.^36, 37^ Therefore, the LumB and HMEC classes were examined to determine whether cell lines lacking a decatenation G_2_ checkpoint exhibit attenuated p53 phosphorylation. To facilitate comparisons across different blots and account for exposure differences, the same NHF1-hTERT sample was loaded onto each gel and the increment of activation of p-Ser15 p53 for each cell line was normalized to that of the NHF1-hTERT internal control. As shown in the upper panel of Fig. 2e, all four HMEC cell lines responded to ICRF-193 with induction of total p53 and p-Ser15 p53. Larger increases in p-Ser15-p53 were observed upon etoposide treatment in the majority of HMEC cell lines, signifying that activation of p53 signaling by ICRF-193 in HMECs is similar to that observed in other non-tumorigenic cell lines.^36^ In contrast, the LumB cell lines exhibited attenuated induction of p-Ser15 p53 in response to ICRF-193 (Fig. 2e, *bottom panel*). At least two of these cell lines (HCC1428 and MDA-MB-453) appeared to express low or undetectable levels of p53 despite harboring wildtype *TP53* genes.^38^ Only the MDA-MB-415 cell line exhibited a mutant form (Y236C) and high basal levels of p53, suggesting that p53 activation was attenuated by a mechanism other than mutation in most of the LumB lines. The increment of p-Ser15 p53 upon etoposide treatment for the LumB class was not statistically different from the HMEC class (Fig. S1B), suggesting that attenuated signaling was not due to the loss of an inherent ability to phosphorylate Ser15 of p53, but rather was less responsive to topo II catalytic inhibition.

Activation of p-Ser 1981 of ATM and p-Thr 68 of Chk2 can contribute to p-Ser15 activation of p53 and have also been observed upon ICRF-193 exposure;^36^ however, no significant differences in ATM or Chk2 activation were observed in the LumB class when compared to the HMEC class, likely due to high levels of variation among cell lines (Fig. S1A–B). Western blots of all cell lines comprising the remaining intrinsic subtypes are shown in Fig. S1C-G. None of the intrinsic subtypes exhibited a statistically significant decrease in ATM or Chk2 when compared to the HMEC class upon either ICRF-193 or etoposide exposure (Fig. S1B); however, the Her2E class also exhibited attenuated activation of p53 in response to ICRF-193. Due to the short exposure time (3 h), this may be a result of the slower growth rate of the Her2E lines as described further below. Taken together, these results indicate that the LumB class harbors a defective decatenation G_2_ checkpoint.

### Breast cancer cell lines exhibit altered S/G_2_/M cell cycle progression

During the initial G_2_/M checkpoint screen, several cell lines exhibited a low MER (Table S1). Because interpretation of checkpoint function can be confounded by differential growth rates, the cell line panel was assessed for differential patterns of cell proliferation and/or deregulated cell cycle progression kinetics to account for inherent differences in cell cycle phase length that might interfere with interpretation of the G_2_/M checkpoint functionality experiments and to ensure that the LumB defect in decatenation G_2_ checkpoint function was not attributable to inherent differences in MER. Such confounding effects have been previously reported and are often due to the dependency of some checkpoint assays on singular markers of cell proliferation.^36^

**Fig. 3:**
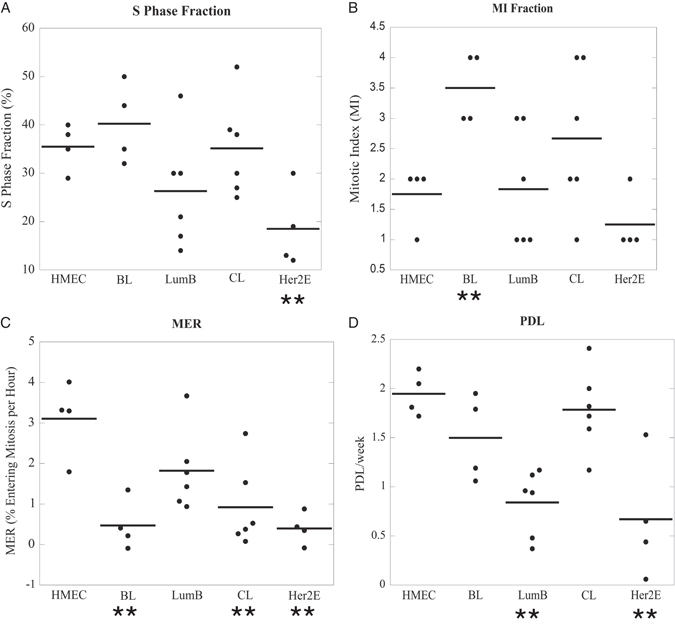
S/G_2_/M progression is aberrantly regulated in breast cancer cell lines of all classes. The average percentage of cells in S phase **(a)** or mitosis **(b)** and the MER **(c)** is shown for each cell line and grouped according to intrinsic molecular subtype. Each point represents the average of at least three independent experiments for an individual cell line, and the bold lines represent the class average. The Her2E class exhibited a lower S phase fraction, the BL class demonstrated a higher MI, and the BL, CL, and Her2E classes displayed lower MERs when compared to the HMEC class. **d** The population doubling level (PDL) over at least 11 weeks in cell culture was determined for each cell line, and the bold lines represent the class averages; both the LumB and Her2E classes exhibited low PDLs. *p-value < 0.05, **p-value that remains significant when controlling for false discovery rate (5%)

Three measures of cell proliferation were compared via flow cytometry including the S phase fraction as determined by EdU incorporation (S), the mitotic index (MI) as measured by MPM2^+^/4N DNA content, and the MER. The number of population doublings (PDLs) occurring during continuous culture was also determined by counting the total number of cells during each passage and calculating the doubling time of each cell line (PDL/week). In Fig. 3 a–d, the individual averages for each cell line are depicted and grouped by molecular subtype. (Averages for each cell line ± SEM are provided in Table S2).

In Fig. 3a, the S phase fractions of the Her2E and LumB subtypes appeared lower than the other classes. LMM analysis supported the observation of a decrease in S for the Her2E class, but the decrease in LumB lines was not statistically significant suggesting that the HMEC, BL, CL, and LumB classes exhibit comparable percentages of cells undergoing DNA replication. In Fig. 3b, the BL cell lines exhibited an increased MI compared to the HMECs, and LMM analysis reinforced this observation suggesting that the HMEC, CL, LumB, and Her2E classes exhibit similar MIs. In Fig. 3c, the MER of the BL, CL, and Her2E classes were lower than the HMEC class; these observations were corroborated by LMM analysis and suggest that only the HMEC and LumB classes exhibited similar MERs. In Fig. 3d, PDL measurements suggested that the LumB and Her2E subtypes have lower PDLs, and LMM analysis validated that both classes exhibited significantly lower PDLs when compared to the HMEC class. The LumB class was comparable to the HMEC class by every other measure of cell proliferation, indicating that a MER comparison of the HMEC and LumB classes was an appropriate measure of both decatenation and DNA damage G_2_ checkpoint function in LumB cell lines. (**p < 0.05, FDR < 0.05)

Because the completion of DNA replication is coupled to the onset of mitosis, concomitant increases in multiple measures of cell proliferation (Fig. 3a–c) are expected to occur in cells that maintain strict regulatory control over S/G_2_/M cell cycle progression to preserve a diploid genome.^39, 40^ Conversely, this relationship would be disrupted if cell cycle phase transitions were deregulated. Thus, two adjacent markers of cell cycle phase were assessed for the presence of a correlative relationship,^41^ and an absence of correlation between those markers would imply that the transition between those two cell cycle phases was delayed and/or aberrantly regulated.

The HMEC and LumB classes exhibited a highly correlative relationship between S phase fraction and MI (*p* < 0.0001 and 0.0041, respectively, Table 1), suggesting that the cell cycle transitions among S/G_2_/M phases follows an ordered progression. However, the BL, CL, and Her2E classes exhibited no correlative relationship between S phase fraction and MI suggesting that cell cycle progression is delayed or aberrantly regulated at some point between the initiation of DNA replication and mitotic exit. To further refine the deregulated point(s) in the cell cycle, S phase fraction was compared to the MER measurement to determine if S or G_2_ progression was delayed. Only the HMEC class (*p* = 0.0006) exhibited a correlation between the S and MER; all of the breast cancer cell line classes displayed decoupling of the S phase fraction and MER suggesting that these classes may spend longer periods of time in the S or G_2_ phases, or exhibit aberrant cell cycle control during S/G_2_. Finally, the HMEC, LumB, and CL classes all exhibited a correlative relationship between the time-dependent MER and MI (*p* = 0.012, *p* < 0.0001, and *p* = 0.0051, respectively) suggesting that these classes maintain ordered mitotic progression. In contrast, the BL and Her2E classes showed no significant correlation between MER and MI, indicating that these classes may not respond to the microtubule polymerization inhibitor colcemid and/or exhibit difficulty completing mitosis. (*p < 0.05, **p < 0.05, FDR < 0.05)

**Table 1:** Predictive relationships between cell cycle growth phases based on cell proliferation markers

Cell line class	S:MI	S:MER	MER:MI	Significant predictive relationships
HMEC	<0.0001**	0.006**	0.0120*	S→MER, MER→MI, and S→MI
BL	0.5996	0.5954	0.1659	N/A
LumB	0.0041*	0.4769	<0.0001**	MER→MI, S→MI
CL	0.4827	0.3758	0.0051*	MER→MI
Her2E	0.0706	0.3090	0.1309	N/A

Breast cancer cell lines exhibit impaired cell cycle regulation and S/G_2_/M progression kinetics*.* Associations between the S:MI, S:MER, and MI:MER growth parameters were assessed for each class. Significant correlations are shown in the right column. Only the HMEC class showed significant associations among all three parameters; all breast cancer cell line classes exhibited aberrant progression kinetics for at least one cell cycle phase transition. *p-value < 0.05, **p-value that remains significant when controlling for false discovery rate (5%)

Only the non-tumorigenic HMEC class exhibited a correlative relationship among all three proliferation markers, suggesting that regulation of the cell cycle in HMECs is linked, occurs in an ordered progression, and illustrates the tightly controlled regulation of S/G_2_/M progression kinetics in non-tumorigenic cells. In contrast, breast cancer cell lines exhibited deregulation of one or more distinct cell cycle phases suggesting that S/G_2_/M progression was delayed or altered. The BL and Her2E classes displayed no correlative relationships, suggesting that multiple defects in cell cycle regulation were present. Because the data in Fig. 3a–d demonstrate that the Her2E class also exhibited lower proliferation rates in culture when compared to the HMECs, we are reluctant to make conclusions regarding cell cycle checkpoint function in cell lines of the Her2E class without additional data. In summary, these data suggest that breast cancer subtypes are associated with altered regulation of S/G_2_/M progression, and that the BL and CL classes may exhibit S/G_2_/M progression defects that evaded detection by the MER assay.

### CL and BL breast cancer cell lines harbor chromatid cohesion defects; the SAC is impaired in BL breast cancer cell lines

**Fig. 4:**
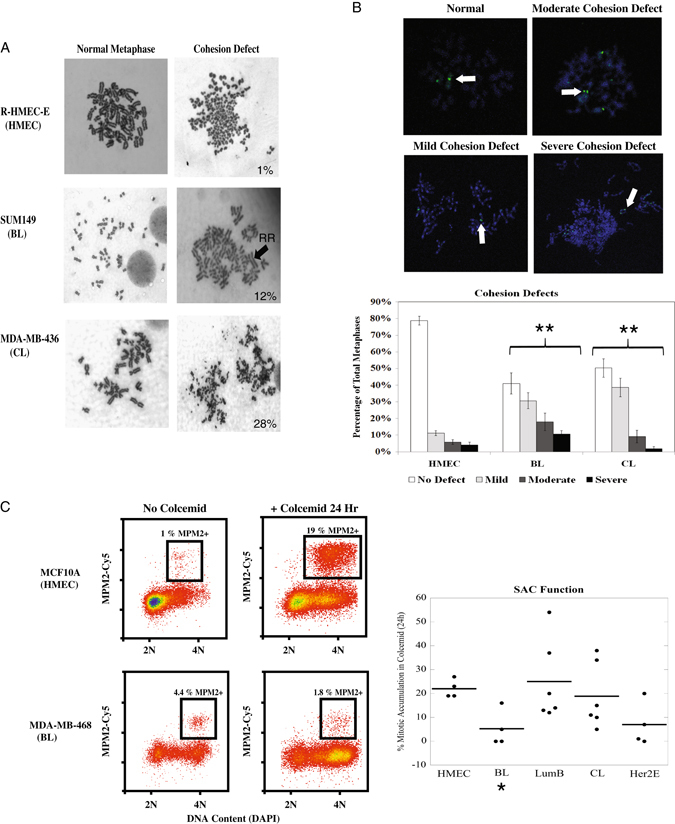
The BL and CL cell line classes exhibit chromatid cohesion defects; the BL class also harbors a defective SAC. **a** Examples of cohesion defects observed in metaphase. The percentages of cells containing cohesion defects is shown for each cell line in the lower right corner of the cohesion defect picture. A “railroad” (RR) chromosome lacking a centromeric constriction point is designated with a *black arrow* in the SUM149 cell line to exemplify a mild cohesion defect. A severe cohesion defect is exemplified by the complete discohesion of the MDA-MB-436 metaphase. **b** Representative examples of the severity of cohesion defects observed during FISH analysis (upper panels). *White arrows* designate the centromere used for cohesion defect classification. Quantification of cohesion defects (*lower panel*) demonstrate that both the BL and CL classes exhibited variation in the distribution of chromatid cohesion defect severity when compared to the HMECs. Results shown were obtained from at least four different biological replicates for each subtype. **c** Flow cytometry examples reflecting SAC function in an HMEC line (MCF10A) and a BL breast cancer line (MDA-MB-468). Individual cell line averages obtained from at least three independent experiments are shown with bold lines representing the class average; the BL class exhibited a decrease in SAC function (*right panel*). *p-value < 0.05, **p-value that remains significant when controlling for false discovery rate (5%)

To determine whether the BL and CL classes exhibited S/G_2_/M checkpoint defects that were not identified by the MER screen, metaphases were analyzed to evaluate gross chromosomal structure aberrations which are often indicative of checkpoint defects. All metaphases were scored in a blind manner, and example spreads are shown in Fig. 4a for an HMEC, BL, and CL cell line. Overall, metaphases of the LumB, and Her2E classes exhibited similar probabilities of harboring a cohesion defect when compared to the HMEC class (Table S3A, *p* = 0.1674 and *p* = 0.4743, respectively). In contrast, the CL and BL classes appeared to exhibit an increased probability of harboring cohesion defects that ranged from mild to severe (Fig. 4a).

Due to the variable range of cohesion defect severity, metaphases were examined via a high sensitivity fluorescence in situ hybridization (FISH) assay utilizing a probe targeting the centromere of chromosome 9 (CEP9) to confirm the presence of cohesion defects and evaluate severity in the BL and CL classes. In diploid metaphase cells with properly cohered sister chromatids, the centromeres of the sister chromatids are so close together that they appear to be a single CEP9 locus upon FISH staining. However, in cells exhibiting cohesion defects, the centromeric regions of the sister chromatids are separated and exhibit a doublet pattern containing two visually distinct CEP9 loci. Degrees of cohesion defects were defined as mild, moderate, or severe,^42^ and examples are shown in Fig. 4b with white arrows indicating each type of cohesion defect. The BL and CL classes both exhibited increases in the total percentage of cells with cohesion defects, suggesting that these classes may experience difficulty completing DNA replication and/or partitioning sister chromatids equally into daughter cells. Furthermore, distributions of cohesion defect severity in both classes also differed when compared to the HMECs, suggesting that an increased number of moderate/severe cohesion defects were found in these classes (Fig. 4b, *lower panel*). In addition, both the BL and CL classes exhibited significantly higher percentages of cells with aneuploidy when compared to HMECs (>4 CEP9 foci per metaphase), suggesting that cohesion defects may lead to inappropriate segregation of duplicate chromosomes during cytokinesis and confirming the presence of genomic instability in these classes (Fig. S2 and Table S3B).

Because the MERs of the CL and BL classes were low compared to the HMEC class (Fig. 3c), the presence of cohesion defects (Fig. 4a, b) have been previously shown to activate the SAC,^43^ and severe aneuploidy was observed in both classes (Table S5B, Fig. S2), it was possible that the BL and CL classes harbored SAC defects. Thus, the number of mitotic cells that accumulated over a 24 h period in the presence/absence of colcemid were measured by flow cytometry.^19, 44^ This assay allows us to distinguish between cell lines that exhibit a low MER due to slow movement through S and/or G_2_ phase (appreciable levels of mitotic accumulation in the presence of colcemid appear over 24 h) and cell lines that lack a functional SAC (no mitotic accumulation due to an inability to arrest in colcemid). Example flow cytometry profiles for MCF10As (HMEC) and MDA-MB-468s (BL) are shown in the left panel of Fig. 4c. Addition of 100 ng/mL colcemid dramatically increased the number of mitotic cells in the MCF10As, whereas the number of mitotic cells decreased in the MDA-MB-468s (Fig. 4c, *middle panels*). The average increase in mitotic index for each cell line grouped by intrinsic molecular subtype is shown for three independent experiments (*right panel*, Fig. 4c). Individual cell line averages are provided in Table S4. Both the BL and the Her2E classes appeared to exhibit a defective SAC, and LMM analysis confirmed that SAC function was reduced in the BL class. Although one BL cell line (SUM149) exhibited some mitotic accumulation in the presence of colcemid, this result is likely due to the presence of a subpopulation of CL cells found in this cell line.^34^ Taken together, these data suggest that the combined cohesion defects and aberrant SAC function identified in the BL class may contribute to the high levels of genomic instability observed in BL breast cancers. The presence of an effective SAC in the CL class suggests that cohesion defects in these cell lines are capable of activating the SAC and that the severe aneuploidy observed is likely due to an alternative mechanism of genomic instability. (*p < 0.05, **p < 0.05, FDR < 0.05)

### A decatenation G_2_ checkpoint gene expression signature correlates with clinical outcomes of breast cancer patients

**Fig. 5:**
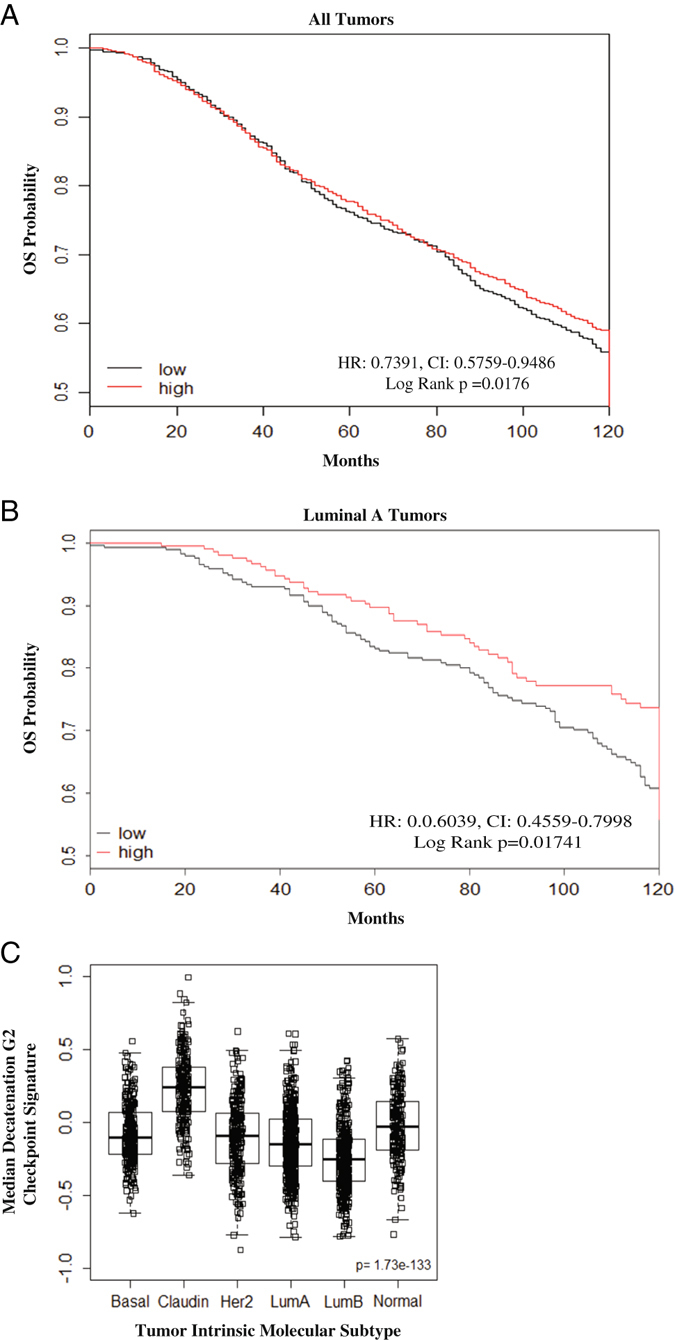
A decatenation G_2_ checkpoint gene expression signature correlates with clinical outcomes of breast cancer patients. **a** gene expression signature that positively correlated with decatenation G_2_ checkpoint function in the breast cancer cell line panel was identified and its relationship with clinical outcomes in the METABRIC study was assessed using a Cox proportional hazards model. A high expression of the decatenation G_2_ checkpoint signature was associated with better overall survival (OS) in all breast tumors (log rank *p* = 0.0176). **b** For the LumA subtype of breast tumors, high expression of the decatenation G_2_ checkpoint was associated with better OS outcomes (Log Rank *p* = 0.01741). **c** Median decatenation G_2_ checkpoint signature varies with intrinsic molecular subtype; LumA and LumB breast tumors exhibited the lowest median expression of the decatenation G_2_ checkpoint signature. HR: hazard ratio, OS: overall survival, CI: confidence intervals

To determine whether the checkpoint defects identified in the cell line panel were associated with clinical outcomes, a gene expression signature of decatenation G_2_ checkpoint function was identified from the cell line panel using quantitative trait analysis (QTA) (Table S5). This signature was applied to the METABRIC clinical data set, which contains approximately 2000 breast tumor samples that include clinical annotations, intrinsic molecular subtyping information, and gene expression data.^45^ In a univariate analysis, the decatenation G_2_ checkpoint signature was significantly associated with better overall survival in all breast tumors (HR: 0.7391, CI: 0.5759–0.9486, log rank *p* = 0.0176, Fig. 5a). In a multivariate analysis including the intrinsic molecular subtypes, the checkpoint signature was not significantly associated with overall survival (HR: 0.76843, CI: 0.5769–1.023, log rank *p* = 0.071673), but was close to significant.

The decatenation G_2_ checkpoint signature was also predictive of OS outcomes in LumA patients (HR: 0.6039, CI: 0.4559–0.7998, *p* = 0.01741, Fig. 5b), suggesting that impaired decatenation G_2_ checkpoint function may contribute to poor outcomes in the LumA subtype. The median decatenation G_2_ checkpoint signature for all breast tumors varied among the subtypes (*p* = 1.73e–133, Fig. 5c) with the LumB subtype expressing the lowest median checkpoint signature; this observation was independently verified using the UNC337 data set (*p* = 8.46e–20, Table S6).^27^ Although the decatenation G_2_ checkpoint signature was not significantly associated with outcomes within the LumB subtype (*p* = 0.597, Table S6), this may be due to the inherently low median decatenation G_2_ checkpoint signature within this subtype. For example, if the majority of LumB tumors are defective for decatenation G_2_ checkpoint function, there may not be enough variation of the signature within the LumB subtype to predict outcomes. Taken together, these data imply that the decatenation G_2_ checkpoint may be a clinically relevant target for the treatment of LumA and/or LumB breast cancer patients.

## Discussion

Although several studies have focused on cell cycle control of human breast cancers and breast cancer cell lines at the G_1_/S transition, only a few have examined G_2_/M checkpoints in multiple cell lines.^46, 47^ However, these studies didn’t address DNA damage G_2_ or decatenation G_2_ checkpoint function, nor did they consider the intrinsic molecular subtype of the cell lines. Therefore, the well-characterized panel of breast cancer and HMEC cell lines described in this work provides a valuable resource summarizing cell cycle checkpoint deregulation and genomic instability patterns observed in a breast cancer cell line model system (Table 2).

**Table 2:** Summary of subtype associated genomic instability phenotypes

Intrinsic molecular subtype	In vitro cell line genomic instability patterns
LumB	•Defective decatenation G2 checkpoint•Functional DNA damage G2 checkpoint•Attenuated p-Ser15 p53 activation in response to catalytic topo II inhibitor•Low PDL•Delayed progression of S or G2 phase•Aneuploidy•Functional SAC
BL	•High MI, but low MER•Aberrant regulation of S/G2/M progression•Aneuploidy•Increased percentages of cohesion defects•Defective SAC
CL	•Low MER•Delayed progression of S and/or G2 phase•Aneuploidy•Increased percentages of cohesion defects•Functional SAC
Her2E	•Attenuated p-Ser15 p53 activation in response to catalytic topo II inhibitor•Low proliferation levels (S, MER, PDL)•Aberrant regulation of S/G2/M progression•Aneuploidy

Note: A summary of growth characteristics, G_2_/M checkpoint function, and genomic instability patterns associated with each intrinsic subtype of breast cancer cell lines

The results of this study indicate that intrinsic subtypes are associated with unique combinations of genomic instability patterns including aberrant cell cycle progression kinetics, chromosomal aberrations, and defective G_2_ or M checkpoints. The initial G_2_/M checkpoint screen revealed that the LumB class exhibited a defect in decatenation G_2_ checkpoint function while remaining capable of activating the DNA damage G_2_ checkpoint (Fig. 2b, 2c), and the results in Fig. 3c and Table 1 support the use of the MER assay as an appropriate measure of G_2_ checkpoint function in LumB cell lines. The defect in decatenation G_2_ checkpoint function was observed in concordance with an attenuation of p-Ser15-p53 upon catalytic topo II inhibition with ICRF-193 (Fig. 2e). Although the rate of *TP53* mutation in primary LumB breast tumors is approximately 30%,^48^ most of the LumB cell lines examined harbored wild-type *TP53* (Table S7), but failed to significantly activate p53 upon ICRF-193 treatment while retaining at least partial p53 activation in response to etoposide. These results suggest that although both drugs share the same target (topo II), they elicit distinct signaling responses in the LumB class and provide further support for the G_2_ checkpoint results (Fig. 1b).

Decatenation G_2_ checkpoint defects have also been observed in lung, bladder, melanoma, and colon cancer cell lines.^37, 49–51^ Two reports have also demonstrated that decatenation G_2_ checkpoint-defective melanoma and colon cancer cell lines are hypersensitive to PI3-kinase inhibitors and topo II catalytic inhibitors, respectively.^51, 52^ Because the decatenation G_2_ checkpoint response is molecularly distinct from the DNA damage G_2_ checkpoint and the SAC,^1, 16, 17^ and the gene expression signature of decatenation G_2_ checkpoint function identified in this study is associated with OS outcomes in breast cancer patients (Fig. 5, Table S6), it may be feasible to induce synthetic lethality in tumors exhibiting defects in decatenation G_2_ checkpoint function with pharmacological agents that catalytically inhibit topo II activity, providing an alternative strategy for targeted drug design to treat poor prognosis LumB breast cancers and a subset of LumA breast cancers. Potential agents that may enhance the cytotoxicity of decatenation G_2_ checkpoint-defective tumor cells include dexrazoxane, an FDA-approved topo II catalytic inhibitor currently used to prevent anthracycline-induced cardiotoxicity, and PI3 kinase inhibitors that are currently in clinical trials for multiple solid tumors.

The low MERs of the BL and CL classes (Table S2 and Fig. 3c) and insignificant correlations between S:MER and MER:MI (Table 1) prompted the examination of metaphases to assess underlying mechanisms that might lead to altered S/G_2_/M progression. Both classes displayed increased levels of cohesion defects and aneuploidy (Fig. 4 and Fig. S2); however, only the BL class failed to activate a SAC response (Fig. 4c). Although a recent report has suggested that two SAC markers are activated in response to Docetaxel in BL cell lines, functional checkpoint assays were not performed suggesting that aberrant activation of those SAC markers may not be the underlying cause of SAC dysfunction.^53^ The significance of these results is further underscored by studies demonstrating that an effective SAC is required for both paclitaxel and anthracyclines to exert cytotoxic effects, and that in the absence of an effective SAC these drugs induce ploidy increases.^22, 23^ These data suggest that the high incidence of cohesion defects coupled with a defective SAC may promote genomic instability, contribute to high relapse rates, and represent a potential synthetic lethal target for BL breast tumors.^22, 23, 43, 54^

Similar to the BL class, the CL cell lines exhibited a lower MER (Fig. 2c) and an increase in the presence of cohesion defects; however, the CL class maintained an intact SAC (Fig. 3a, b) suggesting that an alternative mechanism of genomic instability may be present in this class of cell lines. Curiously, the CL class exhibited similar PDL levels, S, MI, and SAC function in comparison to HMECs (Fig. 3a–d, 4d); however, the lower MERs of this class suggest that these cells may transit through S or G_2_ at a slower rate. Although it is presently unclear what effects sister chromatid cohesion defects may have in the CL subtype, similar mild cohesion defects are associated with genomic instability and the inactivation of MRE-11, SMC1, and CDC4/FBXW7 in colorectal cancers^42^ and future studies will attempt to further characterize S/G_2_/M cell cycle progression defects in the CL subtype.

Components of the cell cycle regulatory machinery and checkpoint function affect the response to chemotherapy in breast cancer cells.^24^ Due to the increasing utilization of breast cancer intrinsic molecular subtypes in clinical settings for prognostic purposes and chemotherapeutic treatment guidance, the identification of subtype-associated genomic instability trends in a standardized panel of in vitro cell lines may contribute to the development of diagnostic assays, novel targeted drug therapies, and new personalized chemotherapy regimens. For example, drugs that target aberrant cell cycle control at the G_0_/G_1_ restriction point such as the cdk4/6 inhibitor Palbociclib® have recently been approved by the FDA to treat hormone therapy-resistant ER^+^ tumors and are currently being exploited in the clinic to induce synthetic lethality in cancers exhibiting deregulation of the RB/cyclin D1 pathway.^25^ Although we attempted to identify subtype-specific genetic mutations in the breast cancer cell line panel that might contribute to the observed genomic instability patterns and identify potential synthetic lethal targets, the resulting data analysis failed to reveal subtype-specific mutations (Fig. S3 and Table S8), similar to published results for primary breast tumors.^48^

The significance of the subtype-associated genomic instability mechanisms outlined herein is further enhanced by the observation that intrinsic molecular subtypes can often indicate an intermediate phenotype; thus, the use of functional G_2_/M checkpoint assays or other measures of genomic instability to complement current clinical determinants of chemotherapeutic response may help relieve some of the ambiguity surrounding these “borderline” tumors. We speculate that hetreogeneous activation of cell cycle checkpoints would likely result in stochastic bypass of these checkpoints leading to inappropriate initiation of DNA synthesis and/or precocious entry into mitosis, similar to the stochastic activation of signaling networks that can promote heterogeneity in tumor cells and stem cells.^55–57^ Such heterogeneity in cell cycle checkpoint activation may affect chemotherapy response, induce a hyper-mutagenic tumor microenvironment, and/or possibly contribute to cancer stem cell maintenance.

This is the first report to identify intrinsic subtype-associated cell cycle checkpoint deregulation in breast cancer cell lines and a potentially clinically relevant role for the decatenation G_2_ checkpoint. In addition, this work has provided a summary of S/G_2_/M progression kinetics, genomic instability mechanisms, and G_2_/M checkpoint defects associated with breast cancer subtypes in a large cell line panel; these characteristics are often difficult to predict by gene expression data alone due to the highly dynamic nature of checkpoints, which often rely on kinase/phosphatase activity and protein degradation pathways to initiate/sustain checkpoint activation. In summary, the subtype-associated genomic instability phenotypes described in this work may be useful for the development of new targeted therapies for the LumB, BL, and CL subtypes, may help identify biomarkers to serve as predictive indicators of therapeutic response, and may explain the underlying causes of differential patient responses to current chemotherapeutic regimens.

## Methods

### Cell line panel

At least four cell lines representing each of the intrinsic molecular subtypes were assayed. Growth medium, conditions, and cell line source are detailed in Table S7. Periodic tests for mycoplasma contamination using a commercial kit were negative. PAM50 subtype calls were based on microarray gene expression data.^34^

### Cell proliferation markers

Cells were plated (day 0), fed (day 2), and treated (day 3) with 10 µM EdU for 2 h and fixed. Samples were stained with: MPM2-Cy5, Click-It EdU-Alexa 488 substrate, and DAPI (See Table S9 for catalog numbers). All samples were analyzed using a Beckman Coulter Dako CyAn ADP Analyzer at the UNC Flow Cytometry Core Facility. At least 30,000 cells were analyzed per sample and percentages of mitotic cells (MPM2^+^/4 N DNA content) or EdU^+^ cells (S phase) were quantified. Results were obtained from at least three independent experiments for each cell line.

### Population doubling level (PDL)

Cells were passaged approximately once a week and fed every 2–3 days with fresh medium. The total number of cells recovered was determined using a Beckman Coulter Counter Z1 instrument and PDL was calculated as follows: PDL = [log(total number of cells recovered/total number of cells plated)]/log2. PDL/week was calculated by determining the number of PDLs occurring over an average 7 day period.

### Flow cytometry MER assay

On day 3, cells were treated with 100 ng/mL colcemid and 0.1% DMSO, 4 µM ICRF-193, or 4 µM etoposide for 0, 2, 4, and 6 h. MPM2-Cy5/DAPI staining of mitotic cells was performed. The rate of change in the percent of cells in mitosis was calculated by regression of the linear portion of the resulting line (2–6 h time points) and the slope of that line was used to quantify MER (% of cells entering mitosis/h) as previously described.^36^ Results were obtained from at least three independent experiments for each cell line.

### Western immunoblotting

Standard immunoblotting techniques were used to detect phosphorylated and total proteins as previously described.^1^ Total and phosphorylated proteins were semi-quantified using Image J software v. 1.45 (NIH) (Table S9—antibody list). Pixel intensities of each phosphorylated protein were normalized to α-tubulin. The increment of increase in protein levels for the drug-treated samples compared to DMSO samples were used to measure protein activation. Values were subsequently normalized to the increment measured for the same NHF1-hTERT positive control sample as an internal normalization control to facilitate comparisons among different western blots and account for different exposures. Western blots shown are representative of two independent experiments.

### Spindle assembly checkpoint assay

Cells were incubated with 100 ng/mL colcemid or 0.1% DMSO (vehicle control) for 0 or 24 h and then fixed, stained, and quantified as described for the MER assay. Results were obtained from at least three independent experiments for each cell line.

### Cytogenetic studies

Metaphases were prepared as previously described,^1^ and examined using an Olympus BH2 microscope (100X objective) with a SPOT RT camera. At least 100 metaphases were scored for each treatment group/cell line when available—if fewer than 100 metaphase cells were present, all metaphases present were analyzed. All analyses were performed blinded to sample identity.

### Fluorescence in situ hybridization (FISH)

FISH samples were prepared as previously described.^1^ At least 100 metaphases were scored per cell line when available—if fewer than 100 metaphase cells were present, all metaphases present were analyzed. All analyses were performed blinded to sample identity on a Zeiss LSM 700 Confocal Laser Scanning Microscope (UNC-CH Microscopy Services Laboratory). The severity of cohesion defects was assessed based on a previous study.^42^ Metaphases containing more than one type of cohesion defect were categorized as exhibiting the most severe cohesion defect.

### QTA and clinical outcomes analysis

Cell line gene expression data were obtained from Dr. Katherine Hoadley and Dr. Charles M. Perou at the University of North Carolina—Chapel Hill and is publicly available.^34^ Array data were Lowess normalized and expressed as log2 ratios. Of the 24 cell lines comprising the panel, 22 were present in the microarray dataset (R-HMEC-E and BT20 were unavailable). QTA was performed using the significance analysis of microarrays (SAM) package in R.^58^ Genes that significantly correlated with a quantitative trait (such as % Inhibition of MER in the presence of ICRF-193 to represent decatenation G_2_ checkpoint function) were selected to generate the gene signature using the QTA tool provided within the SAM package at a low FDR (≤0.1).^59^ These gene lists were subsequently applied to an independent set of publically available clinical data containing overall survival data, clinical annotations, PAM50 subtyping assignments, and gene expression data for approximately 2000 breast cancer patients.^45^ Clinical outcome association analyses were performed using a Cox proportional hazards model in the R survival package.

### Statistical analyses

A set of LMMs were developed to compare the intrinsic subtype classes with the HMEC class. All of the results presented in this manuscript are supported by sensitivity analyses performed using the same data set after removal of one or more highly influential data points (based on the well-known Cook’s distance metric). If the statistical conclusions were the same in both the primary and sensitivity analyses, it was concluded that the particular observation did not unduly influence the analysis and the primary analysis result was reported as statistically significant. All *p*-values, 95% confidence intervals, and details regarding the specific statistical models employed for each individual analysis are included in the supplementary information (SI). *p-value < 0.05, **p-value that remains significant when controlling for false discovery rate (5%).

## Electronic supplementary material

Supplementary Figure and Table Legends

Supplementary Figure 1A-D

Supplementary Figure 1E-G

Supplementary Figure 2

Supplementary Figure 3

Supplementary Table 1

Supplementary Table 2

Supplementary Table 3

Supplementary Table 4

Supplementary Table 5

Supplementary Table 6

Supplementary Table 7

Supplementary Table 8

Supplementary Table 9

Supplementary Statistical Information
