# Supplementary material for: Patterns of cell cycle checkpoint deregulation associated with intrinsic molecular subtypes of human breast cancer cells
Source: NPJ Breast Cancer. 2017 Mar 31;3:9. doi: 10.1038/s41523-017-0009-7 (PMC5445620; doi:10.1038/s41523-017-0009-7)
Supplement: Supplementary file 1 — Supplementary Figure and Table Legends [file 41523_2017_9_MOESM1_ESM.docx]

***Supplementary Tables and Figures:***

***Table S1: Mitotic entry rate measurements for individual breast cancer and HMEC cell lines.*** Results were obtained from at least three independent experiments for each cell line. The values presented in this table are original values obtained prior to the log transformation used for statistical analysis.

***Table S2: Cell cycle proliferation measurements for individual breast cancer and HMEC cell lines.*** Results were obtained from at least three independent experiments for each cell line for S, MI, and MER. Results for the PDL were obtained over at least 11 weeks of cell culture.

***Table S3: Cytogenetic metaphase and FISH measurements for individual breast cancer and HMEC cell lines.* A.** The total number of metaphases analyzed and the percentage of metaphases exhibiting each type of abnormality are shown. Metaphase preparations were attempted for all cell lines included in the panel; however, one BL cell line (HCC1937) and one CL cell line (SUM102) yielded no recognizable metaphases upon Geimsa staining. **B.** Because the BL and CL cell lines appeared to harbor cohesion defects, a highly sensitive FISH analysis was performed and the number of metaphases analyzed by FISH including cohesion defect severity are shown in the bottom table. Due to the expensive nature of the FISH probe, only HMEC, BL, and CL cell lines were analyzed for cohesion defect severity. Two CL cell lines (SUM102 and MDA-MB-435S) failed to yield recognizable metaphases during the FISH analysis. N.A. = data not available.

***Table S4: SAC measurements for individual breast cancer and HMEC cell lines.*** Results were obtained from at least three independent experiments for each cell line.

***Table S5: List of genes associated with decatenation G2 checkpoint function in the breast cancer and HMEC cell lines.*** Array data was available for 22 of the 24 cell lines comprising the panel.

***Table S6: Association of overall, recurrence free, or disease-specific survival with a decatenation G_2_ checkpoint gene expression signature generated from the cell line panel.*** The gene expression signature used for this analysis is provided in Table S5. The median value of the 46 gene signature was calculated and a Cox proportional hazard model was executed using the survival package from the R statistical analysis software. OS: overall survival, RFS: recurrence free survival, DSS: disease-specific survival.

***Table S7: Individual cell line PIK3CA and TP53 mutation status, culture medium/conditions, and cell line source.*** Most cell lines were maintained in the indicated medium at 37⁰ C with 5% CO_2_. Cell lines maintained in L15 medium were cultured in the absence of CO_2_. Cell line source is indicated in the right column. *TP53* and *PIK3CA* mutation status of each cell line was obtained from the Sanger COSMIC Cell Line Database or the Broad Institute’s Cancer Cell Line Encyclopedia Database. N.A.: Not available, TCF UNC-CH: UNC Tissue Culture Facility, mt: mutant, wt: wild-type.

***Table S8:*** ***List of subtype-specific mutations identified for 18 of the 24 cell lines.***  Sequencing data was available for 4 of 4 Her2E cell lines, 3 of 4 BL cell lines, 6 of 6 LumB cell lines, and 5 of 6 CL cell lines. No data were available for any of the HMEC cell lines. NKS: no known significance for the mutation.

***Table S9: List of antibodies and reagents used for all western immunoblots and flow cytometry assays.*** A list of all antibodies and flow cytometry staining reagents used in this study. The company and catalog number of each reagent is provided.

***Figure S1: Western immunoblots showing ATM, Chk2, and p53 activation in response to ICRF-193 or etoposide for all cell lines included in the panel.*** A. Blot of the remaining Luminal B cell line DU4475. B. Quantification of p-Ser15 p53 activation upon etoposide treatment and table of p values according to subtype for activation of ATM, Chk2, and p53. C. Full HMEC western blots. D. Full Luminal B western blots. E. Basal-like western blots. F. Claudin-low western blots. G. Her2-enriched western blots.

***Figure S2: Histogram distributions of chromosome counts obtained from metaphase preparations for each cell line class.*** The x0 value represents the number of chromosomes counted per metaphase with the highest observed frequency.

***Figure S3: Histogram distribution of all mutations observed for the 18 cell lines with available sequencing data.*** Overall there weren’t many commonly recurring mutations in the breast cancer cell lines – the vast majority of the mutations occurred in only 1 cell line. The mutations that occurred with high frequency among the cell lines were almost exclusively in large genes, suggesting that these mutation calls are likely an artifact generated by sequencing errors. Note: not all gene names are listed on the x axis of the figure due to space constraints; however, the figure does contain all catalogued mutations for the entire cell line panel.

***Supplementary Materials and Methods:***

*Statistical Analyses Summary:* All data were subjected to rigorous statistical analysis. Briefly, linear mixed models (LMMs) with cell line random effects were used to determine whether the growth and checkpoint parameters for each subtype significantly differed from the HMECs [1]. For the analysis of MER variables, the outcome and continuous covariates were transformed to the log scale prior to analysis. For the cohesion defect data, quasi-likelihood logistic regression models were used to assess differences in the HMEC class and cancer subtype classes with regards to total cohesion defects, breaks, lost centromeres, radials, end-end fusions, and aneuploidy [2]. The odds ratio was used as the basis for inference. The quasi-likelihood approach was chosen to allow for over-dispersion due to heterogeneity within the cell lines in a given class. In addition, generalized logistic regression models were fit to the cohesion defect data which were comprised of counts of mild, moderate, and severe defects. A global test of the null hypothesis that the defect distribution did not differ between the HMEC class and each breast cancer subtype class was performed. For all analyses, raw p-values and false discovery rate adjusted p-values (5%) were calculated [3]. All p-values, 95% confidence intervals, and details regarding the specific statistical models employed for each individual analysis are included in the statistical supplement. *= p-value <0.05, **= p-value that remains significant when controlling for false discovery rate (5%).

*Mutation Analysis:* The Cancer Cell Line Encyclopedia and COSMIC databases were mined to identify subtype-specific mutations in the cell line panel [4]. Mutation data for 18 of the 24 cell lines were available and the number of cell lines containing an identified mutation is shown in Figure S3. The majority of the mutations identified in the cell lines were single base substitutions, deletions, or insertions. Intrinsic subtype-specific mutations were defined as mutations that were present in at least half of the cell lines of that subtype, but less than three cell lines of any other subtype. Table S9 lists the subtype-specific mutations identified for each cell line class.

References:

1. Fitzmaurice, G., N. Laird, and J. Ware, *Applied Longitudinal Analysis*. Wiley Series in Probabillity and Statistics. 2011, New Jersey: John C. Wiley & Sons.

2. Agresti, A., *Categorical Data Analysis*. Wiley Series in Probability and Statistics. 2002, New Jersey: John C. Wiley & Sons.

3. Benjamini, Y. and D. Yekateuli, *The Control of the False Discovery Rate in Multiple Testing under Dependency.* Annals of Statistics, 2001. **29**: p. 1165-1188.

4. Barretina, J., et al., *The Cancer Cell Line Encyclopedia enables predictive modelling of anticancer drug sensitivity.* Nat Cell Biol, 2012. **483**: p. 603-607.
