# Supplementary material for: Patterns of cell cycle checkpoint deregulation associated with intrinsic molecular subtypes of human breast cancer cells
Source: NPJ Breast Cancer. 2017 Mar 31;3:9. doi: 10.1038/s41523-017-0009-7 (PMC5445620; doi:10.1038/s41523-017-0009-7)
Supplement: Supplementary file 10 — Supplementary Table 5 [file 41523_2017_9_MOESM10_ESM.docx]

| Decatenation G2 checkpoint (+) |
| --- |
| FDR=0.09, N=46 |
| BDKRB2 |
| EDEM2 |
| SCO2 |
| DOCK11 |
| LPAR6 |
| THAP4 |
| LRRC41 |
| FBXO32 |
| IFNGR1 |
| YAP1 |
| ATG9A |
| BDKRB1 |
| SAR1A |
| NARS |
| GSTA4 |
| ARSJ |
| PQLC1 |
| C5orf62 |
| CHI3L2 |
| S100A6 |
| PRAF2 |
| B4GALT2 |
| CD99L2 |
| UST |
| VCL |
| B4GALT1 |
| CRTAP |
| PLSCR4 |
| F2R |
| TMBIM1 |
| SCARB2 |
| ENOX1 |
| CCL2 |
| CD70 |
| ILVBL |
| CFH |
| TFE3 |
| PDIA5 |
| FLRT2 |
| Decatenation G2 checkpoint (+) |
| MARCKS |
| MVP |
| NDST1 |
| FAM76A |
| WNT5A |
| SLC12A4 |
| C5orf23 |
