# Supplementary material for: Patterns of cell cycle checkpoint deregulation associated with intrinsic molecular subtypes of human breast cancer cells
Source: NPJ Breast Cancer. 2017 Mar 31;3:9. doi: 10.1038/s41523-017-0009-7 (PMC5445620; doi:10.1038/s41523-017-0009-7)
Supplement: Supplementary file 11 — Supplementary Table 6 [file 41523_2017_9_MOESM11_ESM.docx]

| UNC337 Clinical Data | Hazard Ratio (HR) | P value | 95% Confidence Interval (CI) |
| --- | --- | --- | --- |
|  | Univariate Analysis |  |  |
| OS – All Tumors | 0.9951 | 0.99 | 0.4672-2.12 |
| RFS – All Tumors | 0.8140 | 0.549 | 0.4152-1.596 |
|  | *MultiVariate Analysis* |  |  |
| RFS Basal | 4.1642 | 0.000148 | 1.9929-8.701 |
| RFS Claudin-low | 2.9822 | 0.008502 | 1.3216-6.730 |
| RFS Her2 | 6.5007 | 1.26e-06 | 3.0488-13.861 |
| RFS Luminal B | 2.9615 | 0.004032 | 1.4130-6.207 |
| RFS Normal | 3.1405 | 0.07 | 0.8831-11.168 |
| RFS Checkpoint Signature | 0.7873 | 0.54702 | 0.3615-1.715 |
|  | High vs. Low Signature |  |  |
| RFS | 0.7117 | 0.139 | 0.4537-1.116 |
| OS | 0.7074 | 0.193 | 0.4202-1.191 |
|  | *High, Med, Low Signature* |  |  |
| OS med | 0.6334 | 0.166 | 0.3319-1.209 |
| OS high | 0.8748 | 0.659 | 0.4826-1.586 |
| RFS med | 0.6522 | 0.129 | 0.3758-1.132 |
| RFS high | 0.7799 | 0.352 | 0.4619-1.317 |
|  | Quartiles of Signature |  |  |
| RFS q2 | 0.9303 | 0.8110 | 0.5143-1.682 |
| RFS q3 | 0.5705 | 0.0986 | 0.2931-1.110 |
| RFS q4 | 0.7995 | 0.4527 | 0.4459-1.434 |
| OS q2 | 0.78221 | 0.4836 | 0.3934-1.555 |
| OS q3 | 0.32674 | 0.0152 | 0.1324-0.806 |
| OS q4 | 0.94737 | 0.8654 | 0.5071-1.770 |
|  | *Outcome Predictions RFS* |  |  |
| LumA (12) | 0.8433 | 0.768 | 0.2711-2.623 |
| LumB(16) | 1.1557 | 0.803 | 0.371-3.6 |
| Normal (3) | 0.1518 | 0.125 | 0.01363-1.691 |
| Basal (18) | 0.4308 | 0.138 | 0.1414-1.312 |
| Her2 (16) | 0.9008 | 0.844 | 0.3172-2.558 |
| Claudin-low (10) | 1.1805 | 0.987 | 0.1281-8.094 |
|  | *Are there differences in the decatenation G2 checkpoint signature among subtypes?* |  |  |
| All subtypes |  | 8.46e-20 |  |
| CL vs. All Other Subtypes |  | 1.385e-07 |  |
| All subtypes – CL |  | 1.41e-07 |  |
| No CL, No normal |  | 0.000123 |  |
| LumA vs. LumB |  | 0.0001812 |  |
| Basal vs. LumA |  | 0.001468 |  |
| Basal vs. LumB |  | 0.7372 |  |
|  |  |  |  |

| METABRIC Clinical Data | Hazard Ratio (HR) | P value | 95% Confidence Interval (CI) |
| --- | --- | --- | --- |
|  | Univariate Analysis |  |  |
| OS – All Tumors | 0.7391 | 0.0176 | 0.5759-0.9486 |
| DSS – All Tumors | 0.8641 | 0.361 | 0.6316-1.182 |
|  | *MultiVariate Analysis (Compared to LumA)* |  |  |
| DSS Basal | 2.7258 | 1.87e-12 | 2.0622-3.603 |
| DSS Claudin-low | 1.8952 | 0.00031 | 1.3390-2.683 |
| DSS Her2 | 2.8959 | 4.67e-14 | 2.1966-3.818 |
| DSS Luminal B | 1.8644 | 4.68e-06 | 1.4280-2.434 |
| DSS Normal | 1.2907 | 0.16892 | 0.8973-1.857 |
| DSS Checkpoint Signature | 0.8165 | 0.27458 | 0.5677-1.175 |
|  | *MultiVariate Analysis (Compared to LumA)* |  |  |
| OS Basal | 1.48324 | 0.000246 | 1.204-1.831 |
| OS Claudin-low | 1.09163 | 0.525584 | 0.8327-1.431 |
| OS Her2 | 1.62824 | 4.11E-06 | 1.3232-2.004 |
| OS Luminal B | 1.20230 | 0.057171 | 0.9944-1.454 |
| OS Normal | 0.96201 | 0.770680 | 0.7414-1.248 |
| OS Checkpoint Signature | 0.76843 | 0.071673 | 0.5769-1.023 |
|  | High vs. Low Checkpoint Signature |  |  |
|  |  |  |  |
| DSS | 0.95072 | 0.554 | 0.8042-1.124 |
| OS | 0.85195 | 0.0177 | 0.7463-0.9726 |
|  | *High, Med, Low Checkpoint Signature* |  |  |
| OS med | 0.79516 | 0.00435 | 0.6793-0.9308 |
| OS high | 0.84990 | 0.04623 | 0.7243-0.9973 |
| DSS med | 0.8383 | 0.0866 | 0.6851-1.026 |
| DSS high | 0.9242 | 0.4450 | 0.7550-1.131 |
|  | Quartiles of Checkpoint Signature |  |  |
| DSS q2 | 0.87056 | 0.236 | 0.6921-1.095 |
| DSS q3 | 0.92724 | 0.515 | 0.7385-1.164 |
| DSS q4 | 0.85290 | 0.187 | 0.6735-1.080 |
| OS q2 | 0.95936 | 0.6428 | 0.8051-1.1432 |
| OS q3 | 0.85585 | 0.0951 | 0.7129-1.0275 |
| OS q4 | 0.81318 | 0.0313 | 0.6736-0.9817 |
|  | *Outcome Predictions OS* |  |  |
| LumA (226) | 0.6039 | 0.000436 | 0.4559-0.7998 |
| LumB(214) | 0.9157 | 0.597 | 0.6608-1.269 |
| Normal (77) | 0.7308 | 0.171 | 0.4665-1.145 |
| Basal (142) | 1.2666 | 0.165 | 0.9074-1.768 |
| Her2 (148) | 0.96762 | 0.842 | 0.7007-1.336 |
| Claudin-low (84) | 0.7071 | 0.326 | 0.354-1.413 |
|  | *Outcome Predictions DSS* |  |  |
| LumA (93) | 0.5878 | 0.0188 | 0.3774-0.9156 |
| LumB(137) | 0.7464 | 0.186 | 0.4838-1.152 |
| Normal (43) | 0.93248 | 0.823 | 0.5059-1.719 |
| Basal (107) | 1.2410 | 0.271 | 0.8452-1.822 |
| Her2 (110) | 1.1942 | 0.356 | 0.8194-1.74 |
| Claudin-low (62) | 0.6488 | 0.281 | 0.2953-1.425 |
|  | *Are there differences in the signatures among subtypes?* |  |  |
| All subtypes |  | 1.73e-133 |  |
| CL vs. All Other Subtypes |  | 1.42e-101 |  |
| No CL, No normal |  | 6.17e-26 |  |
| LumA vs. LumB |  | 8.91e-13 |  |
| Basal vs. LumA |  | 5.23e-05 |  |
| Basal vs. LumB |  | 1.29e-24 |  |
|  |  |  |  |
