# Supplementary material for: Patterns of cell cycle checkpoint deregulation associated with intrinsic molecular subtypes of human breast cancer cells
Source: NPJ Breast Cancer. 2017 Mar 31;3:9. doi: 10.1038/s41523-017-0009-7 (PMC5445620; doi:10.1038/s41523-017-0009-7)
Supplement: Supplementary file 13 — Supplementary Table 8 [file 41523_2017_9_MOESM13_ESM.docx]

***Table S8: Subtype specific mutations in the breast cancer cell line panel***

***Subtype Gene Symbol Description***

***# of LumB Cell Lines with Mutation***

***Nature of***

***Mutations***

***Significance of***

***Mutation?***

Androgen receptor,Steroid- hormone txn factor activates

In frame deletion,

LumB AR

androgen-responsive genes 3 54_55 LL>L NKS

Ser/thr protein kinase, Downstream effector of cdc42

Missense SNP,

LumB CDC42BPB

in cytoskeletal reorganization 3 R570Q NKS Calcium/calmodulin

dependent kinase that

phosphorylates regulator

In frame deletion

LumB MYLK

myosin light chains 3 of E1066 NKS

PI3 phosphatase, tumor suppressor, negatively

Missense SNP,

Possibly decreases PTEN phosphatase activity (Han et al,

LumB PTEN

regulates AKT/PKB 3 C136Y Thought to alter txn via

2000 Cancer Res)

alterations of chromatin

In frame deletion

LumB CHD1

structure 4 of P1684 NKS

***** Sequencing data was avaialble for all 6 of the LumB cell lines used in this study*********

***Subtype Gene Symbol Description***

***# of BL Cell Lines with***

***Mutation***

***Nature of***

***Mutations Significance?***

Not sure what it does, relevant

In frame deletion,

Basal-like MN1

to meningioma and AML 2 544_546 QQQ>Q NKS Missense SNP,

Basal-like MYO3B Class III myosin 2 R1104W NKS

Tumor suppressor, negative regulator of the cell cycle, stabilizes constitutive heterochromatin, active form

Missense SNP,

Basal-like RB1

binds E2F1 2 I388S

***** Sequencing data was avaialble for 3 of the 4 BL cell lines used in this study*********

***Subtype Gene Symbol Description***

Claudin-low None Detected

***** Sequencing data was avaialble for 5 of the 6 CL cell lines used in this study (the SUM102 cell line was unavailable)*****

****

***Subtype Gene Symbol Description***

***# of CL Cell Lines with***

***Mutation***

***# of Her2E Cell Lines with Mutation***

***Nature of***

***Mutations Significance?***

***Nature of***

***Mutations Significance?***

Missense SNP,

Her2 ABCA3 Membrane transporter 2 D724E NKS Regulates DSB repair,

decatenation chkpt, genomic

Missense SNP,

Her2 ATM

stability 2 E2468K NKS

Part of SAC, prevents metaphase to anaphase transition by inhibiting APC/C,

Missense SNP,

Her2 BUB1B

localized to the kinetochore 2 S521F NKS

Apoptosis signaling, activates

In frame deletion

Her2 CARD10

NFkB 2 of KE272 NKS Molecular chaperone

responsible for protein

In frame deletion

Her2 HSP90B1

stabilization and folding 2 of E791 NKS Tyrosine kinase activity, cell

surface receptor for

macrophage stimulating

Missense SNP,

Her2 MST1R

protein 2 F400L NKS Ser/thr kinase, role in

electrolyte homeostasis,

proliferation, cell signaling

Missense SNP,

Her2 WNK2

survival 2 E398Q NKS

Possibly role in phosphatidyl

Missense SNP,

Her2 PLEKHA5

inositol binding? 2 R600T NKS

Retinoblastoma-like 2, key regulator of entry into cell division, epigenetic repression of transcription via regulation

Missense SNP,

Her2 RBL2

of histone methyltransferases 2 D392H NKS

Myocyte cytoskeletal

Nonsense

Her2 SPEG

development 2 Mutation, P2255* NKS Transcriptional coactivator of

RBM14, involved in nuclear

Missense SNP,

Her2 SS18

receptor activation 2 D293N

***** Sequencing data was avaialble for all 4 of the HER2E cell lines used in this study*********
