# Supplementary material for: Patterns of cell cycle checkpoint deregulation associated with intrinsic molecular subtypes of human breast cancer cells
Source: NPJ Breast Cancer. 2017 Mar 31;3:9. doi: 10.1038/s41523-017-0009-7 (PMC5445620; doi:10.1038/s41523-017-0009-7)
Supplement: Supplementary file 14 — Supplementary Table 9 [file 41523_2017_9_MOESM14_ESM.docx]

| ***Antibody*** | ***Company and Catalog #*** |
| --- | --- |
| MPM2-Cy5 | Millipore #16-220 |
| Click-It EdU-Alexa 488 Flow Cytometry Assay Kit | ThermoFisher # C10420 |
| p-Ser1981-ATM | Abcam #ab81292 |
| total ATM | Bethyl #A300-135A |
| p-Ser15-p53 | Cell Signaling #9284 |
| total p53, Clone DO-1 | ThermoFisher #MS-187 |
| p-Thr68-Chk2 | Cell Signaling #2661 |
| total Chk2 | BD Biosciences #611570 |
| α-tubulin | Cell Signaling #2144 |
