# Supplementary material for: Patterns of cell cycle checkpoint deregulation associated with intrinsic molecular subtypes of human breast cancer cells
Source: NPJ Breast Cancer. 2017 Mar 31;3:9. doi: 10.1038/s41523-017-0009-7 (PMC5445620; doi:10.1038/s41523-017-0009-7)
Supplement: Supplementary file 15 — Supplementary Statistical Information [file 41523_2017_9_MOESM15_ESM.docx]

Statistical Analysis For

Cancer Checkpoint Study

Contents

Summary of Statistical Analyses ..…………………………………………………………………………………………………………...3

[Basic Terms 4](#_Toc393016029)

[Notes 4](#_Toc393016030)

[Analysis of Mitotic Index and S Phase Fraction 5](#_Toc393016031)

[Available Data & Questions of Interest 5](#_Toc393016032)

[Statistical Methods 5](#_Toc393016033)

[Results 5](#_Toc393016034)

[Sensitivity Analyses 6](#_Toc393016035)

[Analysis of Mitotic Entry Rate 8](#_Toc393016036)

[Available Data & Questions of Interest 8](#_Toc393016037)

[Statistical Methods 8](#_Toc393016038)

[Results (1)-(3) 9](#_Toc393016039)

[Additional Sensitivity Analyses 10](#_Toc393016040)

[Results (4)-(5) 13](#_Toc393016041)

[Sensitivity Analyses 13](#_Toc393016042)

[Analysis of Population Doubling per Week 13](#_Toc393016043)

[Available Data & Questions of Interest 13](#_Toc393016044)

[Statistical Methods 14](#_Toc393016045)

[Results 14](#_Toc393016046)

[Sensitivity Analyses 14](#_Toc393016047)

SAC Function - [Analysis of 24 Hour Mitotic Accumulation 15](#_Toc393016048)

[Available Data & Questions of Interest 15](#_Toc393016049)

[Statistical Methods 15](#_Toc393016050)

[Results 15](#_Toc393016051)

[Sensitivity Analyses 15](#_Toc393016052)

[Analysis of Chromosomal Defects Counts 16](#_Toc393016053)

[Available Data & Questions of Interest 16](#_Toc393016054)

[Statistical Methods 17](#_Toc393016055)

[Results 17](#_Toc393016056)

[Sensitivity Analyses 18](#_Toc393016057)

[Western Blot Fold Change 18](#_Toc393016058)

[Available Data & Questions of Interest 18](#_Toc393016059)

[Statistical Methods 18](#_Toc393016060)

[Results 18](#_Toc393016061)

[Sensitivity Analyses 19](#_Toc393016062)

[Controlling FDR 20](#_Toc393016063)

# Summary of Statistical Analyses

All data were subjected to rigorous statistical analysis. Briefly, linear mixed models (LMMs) were used to determine whether the growth and checkpoint parameters for each subtype significantly differed from the HMECs in order to account for cell line random effects (i.e. unknown sources of error). The LMM is appropriate when data are approximately normally distributed and when measurements are repeated on each experimental unit (here the experimental units are the cell lines). The LMM we used accounted for the fact that repeated measurements on a cell line will likely be more similar than measurements from distinct cell lines within a cancer subtype. Although an alternative strategy would be to simply average the experimental results from each cell line to obtain a single measurement per cell line and inference on means with the LMM (which makes use of the raw data) and a simple linear model (which makes use of cell line means over replicates) will be similar, the latter procedure can mask potential spurious data points due to the preprocessing step (i.e. averaging step). Modeling the raw data with the LMM allows one to directly assess the influence of each experimental result from each cell line (we did this using the well-known Cook’s distance for sensitivity analysis). For all analyses using the LMM, we verified that the assumption of normality held using QQ plots of residuals, among other things. While it is true that characteristics such as mitotic index (MI) are truly discrete, our experimental measurements were based on a sufficient number of cells so as to justify a continuous approximation. (For example, the MI percentage measurement for each data point was based on a total measurement of 30,000 cells per sample/replicate).

- For the analysis of MER variables, the outcome and continuous covariates were transformed to the log scale prior to analysis.
- For the cohesion defect data, quasi-likelihood logistic regression models were used to assess differences in the HMEC class and BL or CL classes with regards to total cohesion defects, breaks, lost centromeres, radials, end-end fusions, and aneuploidy. The odds ratio was used as the basis for inference. The quasi-likelihood approach was chosen to allow for over-dispersion due to heterogeneity within the cell lines in a given class. In addition, generalized logistic regression models were fit to the cohesion defect data which were comprised of counts of mild, moderate, and severe defects. A global test of the null hypothesis that the defect distribution did not differ between the HMEC class and each breast cancer subtype class was performed.
- For all analyses, raw p-values and false discovery rate adjusted p-values (5%) were calculated. All p-values, 95% confidence intervals, and details regarding the specific statistical models employed for each individual analysis are included within the remainder of this document.
- *= p-value <0.05, **= p-value that remains significant when controlling for false discovery rate (5%).

#

# Basic Terms

In what follows, the following terms are used:

1. Cell class refers to a category of similar cell lines. The cell classes in this study are
   1. HMEC (non-tumorigenic immortalized mammary epithelial cells)
   2. Basal-like
   3. Luminal B
   4. Claudin-low
   5. Her2+

The set of (b)-(e) will be referred to as cancer cell classes.

1. A treatment is a generic term used to describe any medium which is differentially applied to cells. Specifically, we will refer to DMSO, ICRF-193, and Etoposide as treatments in the MER analysis. In the 24 mitotic accumulation analysis, we will refer to Colcemid and Nocodazole as treatments.
2. False discovery rate (FDR) refers to the fraction of significant results that will be false on average. By controlling FDR at the 0.05 level, approximately 5% of the significant results will be false on average.

# Notes

1. P-values that are significant at the 0.05 level are highlighted in gray. Refer to the FDR section for a list of p-values remain significant when controlling FDR for the study.

# Analysis of Mitotic Index and S Phase Fraction

### Purpose: To determine whether the percentage of cells in mitosis (mitotic index, MI) or the percentage of cells undergoing DNA replication (S phase, S) in cancer cell classes differed from that of the non-tumorigenic HMEC class. If significant differences are observed, this would suggest that the cancer cell line classes are progressing through the S/G_2_/M phases of the cell cycle at different rates than the HMEC class and may lend insight into which phase(s) of the cell cycle may be lengthened or shortened.

### Statistical Analysis Details:

### Available Data & Questions of Interest

Data were available in the form of percentage of cells in mitosis or S Phase at a single timepoint. For each cell class, samples were available for several cell lines and samples were replicated on each of the cell lines a varying number of times.

Three questions were of interest for these data.

1. Do the cross-sectional percentage of cells in mitosis (Mitotic Index, MI) differ between each of the cancer cell classes and HMEC? (4 hypotheses)
2. Do the cross-sectional percentage of cells in S Phase (S Phase fraction, S) differ between each of the cancer cell classes and HMEC? (4 hypotheses)
3. Is the S Phase fraction at a time point predictive of the Mitotic Index at the same time point for each of the cell classes? (5 hypotheses)

Based on the above, 13 hypotheses were tested.

### Statistical Methods

We fit three linear mixed models (LMMs) to these data and each was designed to address one of the three questions above.

For questions one and two, the model for the mean included a parameter for each cell class. A random intercept for cell line was included to account for positive correlation between the measurements on a given cell line. Separate error variances were estimated for each cell class.

For model three, we included median centered S Phase fraction as a covariate in the model. We allowed the effect of S Phase fraction on Mitotic Index to vary depending on cell class (i.e. S Phase fraction by cell class interaction).

All outcomes were analyzed on the original scale. Log transformations were considered but did not lead to appreciably better model fit.

### Results

Unadjusted Cell Class MI Comparisons

| **Model** | **Basal-like** | **Luminal-B** | **Claudin-low** | **Her2+** |
| --- | --- | --- | --- | --- |
| 1: MI | 1.8106  (0.6569, 2.9643)  p = 0.0025 | 0.3398  (-0.4520, 1.1316)  p = 0.3957 | 0.6954  (-0.1728, 1.5636)  p = 0.1149 | -0.6305  (-1.4698, 0.2088)  p = 0.1389 |
| 2: S | 6.8516  (-6.4272, 20.1304)  p = 0.3076 | -6.8828  (-18.9891, 5.2235)  p = 0.2613 | 1.5764  (-11.2572, 14.4101)  p = 0.8075 | -15.1217  (-28.0839, -2.1594)  p = 0.0228 |

For model 1: MI, estimates and 95% confidences are for the difference in average MI computed as (Cancer Class – HMEC Class). Hence, a positive estimate indicates more cells in mitosis in the cancer cell class on average. The average MI for basal-like cells differs from the average MI for HMEC cells. No other significant differences were observed.

For model 2: S, estimates and 95% confidences are for the difference in average S Phase fraction computed as (Cancer Class – HMEC Class). Hence, a positive estimate indicates more cells in S Phase in the cancer cell class on average. The average S Phase fraction for Her2+ cells differs from the S Phase fraction for HMEC cells. No other significant differences were observed.

Within Cell Class MI & S Associations

| **Model** | **Basal-like** | **Luminal-B** | **Claudin-low** | **Her2+** | **HMEC** |
| --- | --- | --- | --- | --- | --- |
| 3: MI\|S | 0.0252 (-0.0699, 0.1202)  p = 0.5996 | 0.0401 (0.0131, 0.0670)  p = 0.0041 | 0.0134 (-0.0245, 0.0513)  p = 0.4827 | 0.0286 (-0.0025, 0.0597)  p = 0.0706 | 0.0318 (0.0202, 0.0434)  p = <0.0001 |

For model 3: MI|S, estimates and 95% confidences are for the cell type specific effect of S Phase fraction on MI. The estimates are on the additive scale. The association was significant for Luminal B cells and HMEC cells. For Luminal B cancer cells, an increase in S-fraction by $10\%$ is associated with a $0.0401\cdot10 = 0.401\%$ increase in MI on average. All cell class estimates suggest a positive association in general.

### Sensitivity Analyses

In the main analysis, one observation appeared to have substantial impact on the analysis results. For the analysis from model 1: MI and model 3: MI|S, the observation from the BT20 cell line collected on 27OCT2010 had substantial impact on the estimates of the cell class means (based on Cook’s Distance). Accordingly the analysis was repeated without this observation. The following table presents the results of these sensitivity analyses.

Sensitivity Analyses

| **Model** | **Basal-like** | **Luminal-B** | **Claudin-low** | **Her2+** | **HMEC** |
| --- | --- | --- | --- | --- | --- |
| 1: MI | 1.3295 (0.2745, 2.3845)  p = 0.0142 | 0.3441 (-0.5473, 1.2355)  p = 0.4446 | 0.6983 (-0.2584, 1.6550)  p = 0.1503 | -0.6294 (-1.5816, 0.3228)  p = 0.1922 |  |
| 3: MI\|S | 0.0781 (0.0213, 0.1349)  p = 0.0077 | 0.0363 (0.0067, 0.0659)  p = 0.0169 | 0.0110 (-0.0258, 0.0478)  p = 0.5532 | 0.0209 (-0.0168, 0.0585)  p = 0.2726 | 0.0313 (0.0195, 0.0432)  p = <0.0001 |

The results of this sensitivity analysis agree with those from the main analysis for model 1:MI. However, the results from model 3:MI|S would change. Since no significant results from the main analysis lose their significance in the sensitivity analysis, the primary analysis results will be discussed without qualification.

# Analysis of Mitotic Entry Rate

### Purpose: To determine whether the G_2_/M transition was equally inhibited in the cancer cell line classes when compared to the HMEC class in the presence of an activator of the DNA damage G_2_ checkpoint (etoposide) or the decatenation G_2_ checkpoint (ICRF-193). This assay was used to assess G_2_ checkpoint function among the cancer cell line classes.

### Statistical Analysis Details:

### Available Data & Questions of Interest

Data were available in the form of linear rate of change in the percentage of cells in mitosis (Mitotic Entry Rate, MER) over a 6 hour period under three treatments. For each cell class, samples were available for several cell lines and samples were replicated on each of the cell lines a varying number of times.

Five questions were of interest for these data.

1. Does the MER differ between each of the cancer cell classes and HMEC under each of the treatments? (4x3=12 hypotheses)
2. Is the time-zero MI associated with the MER for each cell class (under DMSO)? (5 hypotheses)
3. Is the time-zero S Phase fraction associated with the MER for each cell class (under DMSO)? (5 hypotheses)
4. Does the DNA damage G2 checkpoint response differ between each cancer cell class and HMEC? (Etoposide % change relative to DMSO, 4 hypotheses)
5. Does the decatenation G2 checkpoint response differ between each cancer cell class and HMEC? (ICRF-193 % change relative to DMSO, 4 hypotheses)

Based on the above, 30 hypotheses were tested.

### Statistical Methods

For analysis of questions (1)-(3), due to skewness in the MER, all variables (outcome & covariates) were transformed to the log scale for analysis. Since the MER can be negative, the response used in analysis was

$$MER_{ijk}=\log\left( 0.55\%+\frac{MI_{6}-MI_{0}}{6} \right).$$

The value $0.55\%$ was chosen to be just larger than the smallest observed MER value. Due to this transformation, confidence intervals for differences given below have no biological interpretation. However, statistically significant differences do correspond to differences on the original scale.

To address question (1), separate models were fit for each treatment. In each of the LMMs, the model for the mean included a parameter each cell class. A random intercept for cell line was included to account for positive correlation between the measurements on a given cell line. Separate error variances were estimated for each cell class.

As a sensitivity analysis, we also adjusted for the time-zero MI and S Phase fraction to assess the impact of time-zero differences as potential confounders. These same models were used to address the question of whether or not time-zero MI and S Phase fraction were associated with MER. In these models, the covariate was transformed to the log scale to match the response and was median centered.

For questions (4) and (5), the outcome used was the percent change in MER under Etoposide or ICRF-193 relative to DMSO. Log transformations of the response did not appreciably improved the model fit. Separate models were fit for each percent change outcome. In each of the LMMs, the model for the mean included a parameter for each cell class. A random intercept for cell line was included to account for positive correlation between the measurements on a given cell line. Separate error variances were estimated for each cell class.

### Results (1)-(3)

Cell Class MER Comparisons (Unadjusted)

| **Model**  **(Treatment)** | **Basal-like** | **Luminal-B** | **Claudin-low** | **Her2+** |
| --- | --- | --- | --- | --- |
| 1a: MER  (DMSO) | -1.1651 (-1.7826, -0.5475)  p = 0.0004 | -0.3266 (-0.9111, 0.2579)  p = 0.2688 | -0.9930 (-1.5619, -0.4241)  p = 0.0009 | -1.1479 (-1.7630, -0.5327)  p = 0.0004 |
| 1b: MER  (Etoposide) | -0.4318 (-1.3643, 0.5007)  p = 0.3588 | 0.4033 (-0.0926, 0.8991)  p = 0.1092 | -0.1421 (-0.6269, 0.3428)  p = 0.5607 | -0.0818 (-0.6166, 0.4531)  p = 0.7613 |
| 1c: MER  (ICRF-193) | -0.2141 (-0.7371, 0.3088)  p = 0.4167 | 0.5668 (0.1123, 1.0214)  p = 0.0153 | -0.3150 (-0.7560, 0.1259)  p = 0.1585 | -0.3651 (-0.8378, 0.1077)  p = 0.1280 |

Cell Class MER Comparisons (Adjusted for Time-zero MI)

| **Model**  **(Treatment)** | **Basal-like** | **Luminal-B** | **Claudin-low** | **Her2+** |
| --- | --- | --- | --- | --- |
| 2a: MER  (DMSO) | -1.2217 (-1.8141, -0.6294)  p = 0.0001 | -0.4097 (-0.9516, 0.1321)  p = 0.1358 | -1.0318 (-1.5692, -0.4944)  p = 0.0003 | -1.3225 (-1.9406, -0.7044)  p = <.0001 |
| 2b: MER  (Etoposide) | -0.3097 (-1.2257, 0.6063)  p = 0.5018 | 0.4062 (-0.0902, 0.9027)  p = 0.1070 | -0.1291 (-0.6104, 0.3522)  p = 0.5938 | -0.2113 (-0.7643, 0.3416)  p = 0.4479 |
| 2c: MER  (ICRF-193) | -0.2191 (-0.7697, 0.3315)  p = 0.4295 | 0.5324 (0.0709, 0.9940)  p = 0.0245 | -0.3194 (-0.7784, 0.1396)  p = 0.1691 | -0.4714 (-0.9835, 0.0407)  p = 0.0705 |

Cell Class MER Comparisons (Adjusted for Time-zero S)

| **Model**  **(Treatment)** | **Basal-like** | **Luminal-B** | **Claudin-low** | **Her2+** |
| --- | --- | --- | --- | --- |
| 3a: MER  (DMSO) | -1.2716 (-1.8849, -0.6582)  p = 0.0001 | -0.3676 (-0.9594, 0.2242)  p = 0.2191 | -1.0882 (-1.6458, -0.5307)  p = 0.0002 | -1.2697 (-1.8786, -0.6608)  p = <.0001 |
| 3b: MER  (Etoposide) | -0.4383 (-1.4240, 0.5475)  p = 0.3777 | 0.4262 (-0.0843, 0.9366)  p = 0.1002 | -0.1443 (-0.6316, 0.3431)  p = 0.5563 | -0.1148 (-0.6523, 0.4227)  p = 0.6710 |
| 3c: MER  (ICRF-193) | -0.2528 (-0.7848, 0.2792)  p = 0.3458 | 0.5535 (0.0823, 1.0246)  p = 0.0221 | -0.3489 (-0.7868, 0.0889)  p = 0.1162 | -0.4109 (-0.8831, 0.0614)  p = 0.0870 |

Key Observations:

- We observe consistent results regardless of whether or not the analysis adjusts for baseline MI or S Phase fraction. Hence, the unadjusted results will be discussed without qualification.
- Significant point estimates less than zero indicate smaller MER for the cancer cell class relative to HMEC.
- Luminal B cells appear to behave differently than the other cancer cells. Under ICRF-193, Luminal B cells have significantly higher MER than HMEC cells.

Within Cell Class Associations

| **Model**  **(Treatment)** | **Basal-like** | **Luminal-B** | **Claudin-low** | **Her2+** | **HMEC** |
| --- | --- | --- | --- | --- | --- |
| 4a: MER\|M  (DMSO) | 0.2385 (-0.1015, 0.5784)  p = 0.1659 | 0.5088 (0.3141, 0.7036)  p = <0.0001 | 0.3583 (0.1116, 0.6049)  p = 0.0051 | -0.3033 (-0.6992, 0.0927)  p = 0.1309 | 0.6261 (0.1422, 1.1099)  p = 0.0120 |
| 4b: MER\|S  (DMSO) | 0.1262 (-0.3464, 0.5989)  p = 0.5954 | 0.1456 (-0.2611, 0.5523)  p = 0.4769 | 0.1251 (-0.1551, 0.4052)  p = 0.3758 | -0.1711 (-0.5044, 0.1623)  p = 0.3090 | 0.5363 (0.2380, 0.8345)  p = 0.0006 |

Key Observations:

- - We are able to detect significant associations between MI and MER for Luminal B and Claudin-low cancer cells and HMEC cells
  - We are able to detect significant associations between S Phase fraction and MER for HMEC cells
  - The Her2+ estimates are puzzling in that associations, while not significant, appear negative. This indicates that increasing the S Phase fraction at time zero is associated with a decrease in MER.

### Additional Sensitivity Analyses

In the above analyses, one observation appeared to have substantial impact on the analysis results. For Etoposide related analyses, the observation from the SUM149 cell line collected on 28OCT2008 had substantial impact on the estimates of the cell class means (based on Cook’s Distance). Accordingly the analysis was repeated without this observation. This observation determined the 0.55% additive factor that was used for the transformation to achieve approximate normality. Accordingly the sensitivity analysis used a reduced conversion factor of 0.30 to match the sensitivity analysis dataset.

The following table presents the results of these sensitivity analyses.

Sensitivity Analyses Cell Class MER Comparisons (Unadjusted)

| **Model**  **(Treatment)** | **Basal-like** | **Luminal-B** | **Claudin-low** | **Her2+** |
| --- | --- | --- | --- | --- |
| 1a: MER  (DMSO) | -1.4800 (-2.3069, -0.6530)  p = 0.0007 | -0.3663 (-1.1301, 0.3975)  p = 0.3420 | -1.2200 (-1.9700, -0.4700)  p = 0.0018 | -1.5109 (-2.3820, -0.6398)  p = 0.0009 |
| 1b: MER  (Etoposide) | -0.1962 (-1.2164, 0.8239)  p = 0.7022 | 0.5301 (-0.3363, 1.3965)  p = 0.2263 | -0.2767 (-1.1430, 0.5896)  p = 0.5259 | -0.2662 (-1.2634, 0.7310)  p = 0.5959 |
| 1c: MER  (ICRF-193) | -0.3845 (-1.1507, 0.3816)  p = 0.3200 | 0.7459 (0.1045, 1.3872)  p = 0.0233 | -0.4883 (-1.1241, 0.1474)  p = 0.1299 | -0.8255 (-1.7790, 0.1280)  p = 0.0886 |

Sensitivity Analyses Cell Class MER Comparisons (Adjusted for Time-zero MI)

| **Model**  **(Treatment)** | **Basal-like** | **Luminal-B** | **Claudin-low** | **Her2+** |
| --- | --- | --- | --- | --- |
| 2a: MER  (DMSO) | -1.5556 (-2.3294, -0.7818)  p = 0.0002 | -0.4654 (-1.1550, 0.2242)  p = 0.1823 | -1.2675 (-1.9538, -0.5812)  p = 0.0005 | -1.9801 (-2.9061, -1.0541)  p = <0.0001 |
| 2b: MER  (Etoposide) | -0.1753 (-1.2319, 0.8814)  p = 0.7413 | 0.5547 (-0.3405, 1.4500)  p = 0.2202 | -0.2464 (-1.1324, 0.6395)  p = 0.5802 | -0.7103 (-1.8317, 0.4111)  p = 0.2102 |
| 2c: MER  (ICRF-193) | -0.3851 (-1.1938, 0.4236)  p = 0.3449 | 0.7024 (0.0449, 1.3599)  p = 0.0367 | -0.4930 (-1.1567, 0.1706)  p = 0.1426 | -1.5954 (-2.7828, -0.4080)  p = 0.0093 |

Sensitivity Analyses Cell Class MER Comparisons (Adjusted for Time-zero S)

| **Model**  **(Treatment)** | **Basal-like** | **Luminal-B** | **Claudin-low** | **Her2+** |
| --- | --- | --- | --- | --- |
| 3a: MER  (DMSO) | -1.6083 (-2.4294, -0.7872)  p = 0.0002 | -0.4102 (-1.1776, 0.3572)  p = 0.2895 | -1.3263 (-2.0606, -0.5921)  p = 0.0006 | -1.6420 (-2.5185, -0.7654)  p = 0.0004 |
| 3b: MER  (Etoposide) | -0.0593 (-1.1442, 1.0255)  p = 0.9133 | 0.5502 (-0.4134, 1.5138)  p = 0.2581 | -0.2794 (-1.2302, 0.6713)  p = 0.5590 | -0.3114 (-1.4091, 0.7864)  p = 0.5728 |
| 3c: MER  (ICRF-193) | -0.4349 (-1.2238, 0.3540)  p = 0.2747 | 0.7034 (0.0380, 1.3689)  p = 0.0386 | -0.5406 (-1.1783, 0.0971)  p = 0.0952 | -0.8526 (-1.8626, 0.1573)  p = 0.0965 |

Sensitivity Analyses within Cell Class Associations

| **Model**  **(Treatment)** | **Basal-like** | **Luminal-B** | **Claudin-low** | **Her2+** | **HMEC** |
| --- | --- | --- | --- | --- | --- |
| 4a: MER\|M  (DMSO) | 0.3474 (-0.1826, 0.8773)  p = 0.1950 | 0.6021 (0.3779, 0.8263)  p = <0.0001 | 0.4853 (0.1807, 0.7899)  p = 0.0023 | -0.9007 (-1.8304, 0.0290)  p = 0.0573 | 0.7380 (0.1730, 1.3029)  p = 0.0113 |
| 4b: MER\|S  (DMSO) | 0.2174 (-0.5072, 0.9420)  p = 0.5510 | 0.1698 (-0.3099, 0.6495)  p = 0.4820 | 0.1318 (-0.2308, 0.4944)  p = 0.4702 | -0.1659 (-1.0127, 0.6809)  p = 0.6967 | 0.6141 (0.2584, 0.9699)  p = 0.0010 |

The results appear to be stable. No results that were significant in the main analysis are not significant in the sensitivity analysis. Hence, the main analysis results will be discussed without qualification. Please note that the values themselves cannot be compared between the two sets of analyses since the additive transformation factor is different.

### Results (4)-(5)

Cell Class MER Comparisons

| **Treatment**  **(Outcome)** | **Basal-like** | **Luminal-B** | **Claudin-low** | **Her2+** |
| --- | --- | --- | --- | --- |
| Etoposide  (DNA Damage) | -0.3043 (-1.6409, 1.0323)  p = 0.6510 | -0.2546 (-0.5715, 0.0622)  p = 0.1134 | 0.2087 (-0.1190, 0.5364)  p = 0.2081 | -0.3562 (-0.8974, 0.1850)  p = 0.1935 |
| ICRF-193  (Decatenation) | 0.3108 (-0.8863, 1.5078)  p = 0.6060 | -0.5505 (-0.8647, -0.2363)  p = 0.0008 | 0.0832 (-0.2649, 0.4314)  p = 0.6347 | -0.3013 (-0.8151, 0.2126)  p = 0.2460 |

Key Observations:

- We observe a significant difference in the decatenation checkpoint measure for luminal B cells relative to HMEC cells. No other significant differences were observed.

### Sensitivity Analyses

In these analyses, two observations had substantial impact on the estimates based on Cook’s Distance. The observation from the MDA-MB-468 cell line collected on 27APR2009 and the observation from the UACC812 cell line collected on 31MAR2010 both had near double the impact of any other observation. Accordingly, the analysis was repeated without these observations. The results are included in the following table.

| **Treatment**  **(Outcome)** | **Basal-like** | **Luminal-B** | **Claudin-low** | **Her2+** |
| --- | --- | --- | --- | --- |
| Etoposide  (DNA Damage) | 0.2207 (-0.6344, 1.0758)  p = 0.6079 | -0.2543 (-0.5634, 0.0547)  p = 0.1050 | 0.2084 (-0.1117, 0.5284)  p = 0.1981 | -0.1704 (-0.5408, 0.2000)  p = 0.3617 |
| ICRF-193  (Decatenation) | -0.2148 (-0.8159, 0.3864)  p = 0.4781 | -0.5503 (-0.8532, -0.2474)  p = 0.0006 | 0.0817 (-0.2566, 0.4201)  p = 0.6311 | -0.1406 (-0.5535, 0.2723)  p = 0.4988 |

The results are consistent. We also considered models that adjusted for baseline MI as a potential confounder (not presented) and the results were again consistent with those presented here. The main analysis results will be discussed without qualification.

# Analysis of Population Doubling per Week

### Purpose:

To determine whether the population doubling time of the cancer cell line classes was increased or decreased when compared to the HMEC class. These data are indicative of cellular proliferation rates.

### Statistical Analysis Details:

### Available Data & Questions of Interest

Data were available in the form of number of population doublings (PD) over a variable number of days. For each cell class, samples were available for several cell lines and samples were replicated on each of the cell lines a varying number of times. In addition, a given replicate may be measured multiple times (started, measured, some cells restarted, measured, and so on). Only samples where the net gain in cells over the period was positive and with a growth period larger than 2 days were included in this analysis.

The (log_2_ of the) number of cells at the start of a growth period is available as a covariate. In addition, we constructed several covariates from the available data:

- Short growth period = Indicator of whether the growth period was < 7 days long
- Long growth period = Indicator of whether the growth period was > 21 days long

PD per week is defined as log_2_(end size / start size) / # weeks of growth. The hypotheses tested were that the PD per week did not differ between each cancer cell class and HMEC. Four (4) hypotheses were tested.

### Statistical Methods

We fit a single Linear mixed model (LMM) where the response was the PD per week.

The model for the mean included a parameter for each cell class and was adjusted for the baseline number of cells on the log scale, whether the growth period was short, and whether the growth period was long (see above). A random intercept for cell line was included to account for positive correlation between the measurements on a given cell line. Separate error variances were estimated for each cell class.

Residual plots suggest no transformation of the response was necessary.

### Results

| **Outcome** | **Basal-like** | **Luminal B** | **Claudin-Low** | **Her2+** |
| --- | --- | --- | --- | --- |
| Population Doubling per Week | -0.4942 (-1.0196, 0.0313)  p = 0.0653 | -0.9735 (-1.4656, -0.4815)  p = 0.0001 | -0.2411 (-0.7265, 0.2443)  p = 0.3297 | -1.1576 (-1.6836, -0.6317)  p = <0.0001 |

### Sensitivity Analyses

No diagnostics suggested a sensitivity analysis.

# SAC Function - Analysis of 24 Hour Mitotic Accumulation

### Purpose: To determine whether the cancer cell line classes accumulated in colcemid or nocodazole at levels similar to that of the HMEC class. This assay was used to assess SAC function among the cancer cell line classes.

### Statistical Analysis Details:

### Available Data & Questions of Interest

Data were available in the form of percent of cells in mitosis at baseline and 24 hours after baseline. At baseline, cells were not treated. After baseline, cells were divided and subsets treated with Colcemid or Nocodazole. For each cell class, samples were available for several cell lines and samples were replicated on each of the cell lines a varying number of times.

Mitotic accumulation under a particular treatment is defined as the difference between the 24 hour measurement on that treatment and the baseline measure. The hypotheses tested were that the 24 hour mitotic accumulation under a particular treatment did not differ between each cancer cell class and HMEC.

Eight (8) hypotheses were tested:

- 4 cancer cell classes x 2 treatments = 8 hypotheses

### Statistical Methods

We fit a Linear mixed model (LMM) where the response was the 24 hour mitotic accumulation.

The model for the mean included a parameter for each cell class and treatment combination and adjusted for the baseline percent of cells in mitosis. A random intercept for cell line was included to account for positive correlation between the measurements on a given cell line. Separate error variances were estimated for each cell class.

Residual plots suggest no transformation was necessary.

### Results

| **Treatment** | **Basal-like** | **Luminal-B** | **Claudin-low** | **Her2+** |
| --- | --- | --- | --- | --- |
| Colcemid | 16.9673 (1.8271, 32.1074)  p = 0.0283 | -3.2618 (-17.2045, 10.6809)  p = 0.6444 | 2.4506 (-11.7139, 16.6152)  p = 0.7328 | 11.5838 (-3.7623, 26.9300)  p = 0.1378 |
| Nocodazole | 10.3274 (-5.0941, 25.7489)  p = 0.1876 | -14.0098 (-28.2978, 0.2782)  p = 0.0546 | -2.9848 (-17.5170, 11.5475)  p = 0.6853 | 5.6188 (-9.8664, 21.1040)  p = 0.4743 |

Estimates and confidence intervals are for the average difference in 24 hour mitotic accumulation computed as (HMEC Class - Cancer Class). Hence, a positive estimate indicates more cells accumulated for HMEC on average. Only basal-like cells under Colcemid demonstrated a significant difference relative to HMEC.

### Sensitivity Analyses

No diagnostics suggested a sensitivity analysis.

# Analysis of Chromosomal Defects Counts

### Purpose: To determine whether a metaphase obtained from the cancer cell line classes had a higher probability of exhibiting a specific chromosomal aberration when compared to a metaphase of the HMEC class. In addition, the distribution of cohesion defect severity was analyzed to determine whether the cohesion defects observed in the cancer cell lines were more severe than those observed in the non-tumorigenic HMEC class.

### Statistical Analysis Details:

### Available Data & Questions of Interest

There were two datasets available for this analysis. We refer to them as FISH and non-FISH data. The defects that were examined in the non-FISH analysis were:

1. Chromosomal breaks
2. Cohesion Defects
3. Lost Centromeres
4. End-End Fusions
5. Radials

The defects that were examined in the FISH data analysis were:

1. Aneuploidy
2. Cohesion Defects by severity (No Defect, Mild, Moderate, Severe)

In both cases, the number of cells having a particular defect out of a fixed number of cells examined were counted. Samples were obtained from 2-5 different cell lines in each cell class for the non-FISH data. For FISH data, only Basal-like and Claudin-low cancer cell classes were examined. No experiments were replicated on a given cell line.

The goal of these analyses was to determine if there is statistical evidence that the probability of a cell having a particular defect differs between HMEC and each of the cancer cell classes. For the cohesion defect by severity data, the cancer cell classes were also compared. For the non-FISH data analysis, twenty (20) hypotheses were tested:

- 4 cancer cell classes x 5 defects = 20 hypotheses based on non-FISH data

For the FISH data, five (5) hypotheses were tested:

- 2 cancer cell classes x 2 defects = 4 hypotheses based on non-FISH data
- 1 comparison of the cancer cell classes for cohesion defect by severity data

For all but the cohesion defect by severity analysis, the null hypothesis for each test (i.e. each defect and cancer cell class) was that the probability of a cell having a defect for the cancer cell class was the same as for HMEC. For the cohesion defect by severity analysis, we tested the null hypothesis that the defect severity distribution (i.e. probabilities of having defects in the various severity levels) did not differ between the classes.

### Statistical Methods

#### Binary Defect Measures

Summary:

- Estimation method:
  - Quasi-likelihood (logistic) estimating equations
  - The overdispersion parameter was estimated by the square root of Pearson's Chi-Square/DOF.
- Separate analyses were performed for each defect
- Computed odds ratios (ORs) comparing the odds of a cell having a given defect for each cell class relative to HMEC

#### Cohesion Defect by Severity

Summary:

- Nominal Categories used: No Defect, Mild, Moderate, Severe
- Estimation method:
  - Baseline-Category Logistic Model without overdispersion parameter
  - The overdispersion parameter could not be estimated due to insufficient replication.
- The data demonstrated a transparent violation of the proportional odds model leading to this choice of statistical procedure.

### Results

Binary Outcome Analyses

| **Defect** | **Basal-like** | **Luminal B** | **Claudin-low** | **Her2+** |
| --- | --- | --- | --- | --- |
| Breaks | 1.7314 (0.0167, 179.2173)  p = 0.8166 | 3.7954 (0.1097, 131.3126)  p = 0.4607 | 11.7182 (0.3919, 350.3628)  p = 0.1557 | 33.6667 (1.0629, 1066.4112)  p = 0.0461 |
| Cohesion  Defects | 21.5714 (0.7070, 658.1280)  p = 0.0782 | 10.6667 (0.3703, 307.2802)  p = 0.1674 | 28.1041 (1.0175, 776.2585)  p = 0.0488 | 4.6701 (0.0685, 318.1725)  p = 0.4743 |
| Lost  Centromeres | 1.9746 (0.1558, 25.0292)  p = 0.5996 | 2.6281 (0.3363, 20.5347)  p = 0.3570 | 2.5358 (0.3028, 21.2344)  p = 0.3908 | 7.6886 (0.7777, 76.0140)  p = 0.0810 |
| Aneuploidy | 34.8740 (16.0389, 75.8276)  p = <0.0001 |  | 34.2537 (14.7865, 79.3507)  p = <.0001 |  |

Notes:

1. Radials and End-End fusions had zero defects in the HMEC group. ML estimates do not allow for good comparisons.
2. The above quantities are log(OR) where the OR is computed using the cancer class in the numerator. Hence, estimates that are positive indicate increased probability of the defect in the cancer cell class.

Multivariate Outcome Analyses

| **Defect** | **Basal-like** | **Claudin-low** | **Basal-like vs. Claudin-low** |
| --- | --- | --- | --- |
| Cohesion Defects (Severity) | p = <0.0001 | p = <0.0001 | p = 0.0201 |

In both cases, there is a strong result that the distribution of defect severities differ in both cancer classes relative to HMEC.

### Sensitivity Analyses

No sensitivity analysis was performed.

# Western Blot Fold Change

### Purpose: To compare p53 activation levels among the cancer cell line classes and the HMEC class in the presence of an activator of the DNA damage G_2_ checkpoint or the decatenation G_2_ checkpoint. This assay was used to determine whether defects in the ATM/Chk2/p53 signaling pathway were present in the cancer cell line classes.

### Statistical Analysis Details:

### Available Data & Questions of Interest

Data were available in the form of fold increase in pixel density from Western Blot analysis under Etoposide or ICRF-193 relative to DMSO for all cell classes and for proteins ATM, Chk2, and p53. No replicates on a given cell line were available. The hypotheses tested were that the fold change in pixel density under a given treatment relative to DMSO did not differ between the cancer cell classes and HMEC for each of the proteins above.

Twenty-four (24) hypotheses were tested:

- 3 proteins x 4 cancer cell class x 2 treatments = 24 hypotheses

### Statistical Methods

Due to insufficient replication on the cell Iines used, mixed models could not be considered. Accordingly, two-sample t-tests (with unequal variances) were used. Data were analyzed on the log scale.

### Results

| **Treatment** | **Protein** | **Basal-like** | **Luminal B** | **Claudin-low** | **Her2+** |
| --- | --- | --- | --- | --- | --- |
| Etoposide | ATM | 1.1801 (-0.8639, 3.2240)  p = 0.2408 | 0.8713 (-0.2589, 2.0015)  p = 0.1227 | 1.5697 (0.3495, 2.7900)  p = 0.0146 | 2.3829 (0.4746, 4.2911)  p = 0.0172 |
|  | Chk2 | 0.9085 (-0.6611, 2.4781)  p = 0.2397 | 0.2380 (-1.2219, 1.6980)  p = 0.7359 | 0.2553 (-1.0313, 1.5418)  p = 0.6817 | 1.3242 (0.1365, 2.5119)  p = 0.0309 |
|  | TP53 | 3.3226 (0.3199, 6.3254)  p = 0.0320 | 0.9887 (-0.8433, 2.8206)  p = 0.2717 | 0.2712 (-1.1825, 1.7250)  p = 0.6997 | 0.7697 (-1.5206, 3.0599)  p = 0.4892 |
| ICRF-193 | ATM | 0.5389 (-0.3349, 1.4126)  p = 0.2115 | 0.4562 (-0.0865, 0.9990)  p = 0.0943 | 0.5444 (-0.0397, 1.1286)  p = 0.0659 | 1.0990 (0.0498, 2.1481)  p = 0.0411 |
|  | Chk2 | 0.6329 (-0.1913, 1.4571)  p = 0.1241 | 0.8148 (-0.1660, 1.7956)  p = 0.0980 | 0.0033 (-0.9092, 0.9158)  p = 0.9940 | -0.3904 (-2.2356, 1.4549)  p = 0.6620 |
|  | TP53 | 2.6892 (-0.0861, 5.4645)  p = 0.0568 | 0.9002 (0.4139, 1.3865)  p = 0.0011 | 0.4783 (-0.2793, 1.2360)  p = 0.2013 | 1.1535 (-1.3412, 3.6482)  p = 0.3442 |

Estimates and confidence intervals are for the log(HMEC) – log(cancer cell class) difference in pixel density fold change under the respective treatment relative to DMSO. Hence, a positive estimate indicates greater fold change for HMEC.

### Sensitivity Analyses

No diagnostics suggested a sensitivity analysis.

# Controlling FDR

In this study, a total of 104 hypotheses were tested. The following table presents the raw p-values and the FDR adjusted p-values. We use a conservative FDR adjustment that will control FDR regardless of the dependence between tests. The method used, presented by Benjamini and Yekateuli (2001), is a conservative method. The following table includes the 29 results that where significant at the 0.05 level. Of the 29 results, 14 remain significant when we control FDR at 5%. These results are highlighted in the table below.

| **Test Description** | **Class** | **Raw**  **P-value** | **FDR**  **P-value** | **FDR P-value**  **Significant?** |
| --- | --- | --- | --- | --- |
| 24 Hour Acc. on Colcemid | Basal-like | 0.0283 | 0.6418 | No |
| Defect: Aneuploidy | Basal-like | <.0001 | <.0001 | Yes |
| Defect: Aneuploidy | Claudin-low | <.0001 | <.0001 | Yes |
| Defect: Breaks | Her2+ | 0.0461 | 0.8948 | No |
| Defect: Cohesion Defects | Claudin-low | 0.0488 | 0.9149 | No |
| Defect: Cohesion Defects (Severity) | Basal-like | <.0001 | <.0001 | Yes |
| Defect: Cohesion Defects (Severity) | Basal-like v. Claudin-low | 0.0201 | 0.4972 | No |
| Defect: Cohesion Defects (Severity) | Claudin-low | <.0001 | <.0001 | Yes |
| Fold Change: Etoposide | Basal-like | 0.0320 | 0.6686 | No |
| Fold Change: Etoposide | Claudin-Low | 0.0146 | 0.4159 | No |
| Fold Change: Etoposide | Her2+ | 0.0172 | 0.4459 | No |
| Fold Change: Etoposide | Her2+ | 0.0309 | 0.6686 | No |
| Fold Change: ICRF-193 | Her2+ | 0.0411 | 0.8265 | No |
| Fold Change: ICRF-193 | Luminal B | 0.0011 | 0.0418 | Yes |
| Fraction: MI | Basal-like | 0.0025 | 0.0901 | No |
| Fraction: MI\|S (MI and S association) | HMEC | <.0001 | <.0001 | Yes |
| Fraction: MI\|S (MI and S association) | Luminal B | 0.0041 | 0.1400 | No |
| Fraction: S | Her2+ | 0.0228 | 0.5387 | No |
| MER (DMSO) | Basal-like | 0.0004 | 0.0212 | Yes |
| MER (DMSO) | Claudin-low | 0.0009 | 0.0364 | Yes |
| MER (DMSO) | Her2+ | 0.0004 | 0.0218 | Yes |
| MER (ICRF-193) | Luminal B | 0.0153 | 0.4159 | No |
| MER\|M (DMSO) (MER and MI association) | Claudin-low | 0.0051 | 0.1629 | No |
| MER\|M (DMSO) (MER and MI association) | HMEC | 0.0120 | 0.3636 | No |
| MER\|M (DMSO) (MER and MI association) | Luminal B | <.0001 | 0.0002 | Yes |
| MER\|S (DMSO) (MER and S association) | HMEC | 0.0006 | 0.0317 | Yes |
| Percent Inhibition (ICRF-193) | Luminal B | 0.0008 | 0.0364 | Yes |
| Population Doubling per Week | Her2+ | <.0001 | 0.0014 | Yes |
| Population Doubling per Week | Luminal B | 0.0001 | 0.0077 | Yes |
